# Identifying determinants of persistent MRSA bacteremia using mathematical modeling

**DOI:** 10.1371/journal.pcbi.1007087

**Published:** 2019-07-11

**Authors:** Tsuyoshi Mikkaichi, Michael R. Yeaman, Alexander Hoffmann, MRSA Systems Immunobiology Group

**Affiliations:** 1 Institute for Quantitative and Computational Biosciences, University of California, Los Angeles, California, United States of America; 2 Department of Microbiology, Immunology, and Molecular Genetics, University of California, Los Angeles, California, United States of America; 3 David Geffen School of Medicine at UCLA, Los Angeles, California, United States of America; 4 Divisions of Molecular Medicine and Infectious Diseases, Harbor-UCLA Medical Center, Torrance, California, United States of America; 5 Los Angeles Biomedical Research Institute at Harbor-UCLA Medical Center, Torrance, California, United States of America; Yale School of Public Health, UNITED STATES

## Abstract

Persistent bacteremia caused by *Staphylococcus aureus* (SA), especially methicillin-resistant SA (MRSA), is a significant cause of morbidity and mortality. Despite susceptibility phenotypes *in vitro*, persistent MRSA strains fail to clear with appropriate anti-MRSA therapy during bacteremia *in vivo*. Thus, identifying the factors that cause such MRSA persistence is of direct and urgent clinical relevance. To address the dynamics of MRSA persistence in the face of host immunity and typical antibiotic regimens, we developed a mathematical model based on the overarching assumption that phenotypic heterogeneity is a hallmark of MRSA persistence. First, we applied an ensemble modeling approach and obtained parameter sets that satisfied the condition of a minimum inoculum dose to establish infection. Second, by simulating with the selected parameter sets under vancomycin therapy which follows clinical practices, we distinguished the models resulting in resolving or persistent bacteremia, based on the total SA exceeding a detection limit after five days of treatment. Third, to find key determinants that discriminate resolving and persistent bacteremia, we applied a machine learning approach and found that the immune clearance rate of persister cells is a key feature. But, fourth, when relapsing bacteremia was considered, the growth rate of persister cells was also found to be a key feature. Finally, we explored pharmacological strategies for persistent and relapsing bacteremia and found that a persister killer, but not a persister formation inhibitor, could provide for an effective cure the persistent bacteremia. Thus, to develop better clinical solutions for MRSA persistence and relapse, our modeling results indicate that we need to better understand the pathogen-host interactions of persister MRSAs *in vivo*.

## Author summary

*Staphylococcus aureus* causes potentially lethal infections of the bloodstream and target organs when able to enter the body, often via skin trauma or catheterization. Methicillin-resistant *Staphylococcus aureus* (MRSA) resist common antibiotics, but are often successfully treated with vancomycin. However, in some MRSA patients, vancomycin is less effective. This results in persistent bacteremia, even though the isolates can be effectively killed *in vitro*. MRSA bacteria are thought to switch between two forms, normal and persister cells, that are genetically identical. Persisters, the minor subpopulation, are slow-growing and show lower susceptibility to vancomycin than normal MRSA. To understand the dynamic interplay between the two bacterial populations when challenged by host immunity and vancomycin treatment, we developed a mathematical model and analyzed it in simulations of clinically relevant scenarios. Our work suggests that the immune clearance rate of persister MRSA rather than the MRSA switch rate is a key determinant to establish persistent bacteremia. The model also suggests that increasing killing rate of persisters is a promising therapeutic strategy. Our findings emphasize the need to better understand the interactions of persister MRSAs with host cells and immune responses *in vivo*.

## Introduction

*Staphylococcus aureus* (SA) is one of the most common life-threatening human pathogens [1–3]. Methicillin-resistant strains (MRSA) exhibit high rates of morbidity and mortality [1–3]. MRSA bacteremia may be treated with anti-MRSA antibiotics, such as vancomycin or daptomycin. However, such treatments fail in about 30–50% of patients, resulting in persistent bacteremia [4,5]. Persistent MRSA bacteremia, which is defined as 3–7 days positive blood culture post-therapy [1,6,7], is recognized as an urgent public health concern, as increased duration of bacteremia is associated with poor clinical outcomes, such as metastatic and complicated infections [6,8,9]. There are presently few therapeutic options for treating persistent MRSA bacteremia [1,10].

Although anti-MRSA antibiotics are ineffective against persistent MRSA strains *in vivo*, isolates from such patients have susceptible minimum inhibitory concentration (MIC) breakpoints, which is the lowest concentration that inhibits growth when cultured *in vitro* [1]. Thus, persistence differs from classical resistance, in which isolates exhibit resistance to antibiotics both *in vivo* and *in vitro*. Persistent bacteremia (PB) is attributed to persistent infection, as bacteria detected in blood derive from infection foci in internal organs, and/or endocarditis lesions [1,11–13]. In contrast, in resolving bacteremia (RB), antibiotic treatment leads to a remission of bacteremia within a few days, indicating that MRSA in infection sites was eradicated. Further, when the infection was not cleared, it may then result in relapsing bacteremia when antibiotic treatment is terminated [14,15]. Relatively little is known about the specific genotypic or phenotypic characteristics of SA that result in PB. Hence, there is a critical unmet need to understand the bacterial, host, and/or antibiotic factors that contribute to PB.

The phenomenon of bacterial persistence was first reported in the 1940’s [16,17] and, until now, it has been observed for several pathogens such as SA, *Mycobacterium tuberculosis*, *Salmonella*, *Escherichia coli* (*E. coli*) [18]. When cultured *in vitro*, most of the bacteria are killed efficiently by antibiotics, but a small portion survives and can regrow after removing antibiotics. As opposed to resistance, the regrown descendants are sensitive to antibiotics [19]. Bacterial persistence may be explained by a phenotypic heterogeneity within the bacterial population in which at least two distinct types of cells, genetically identical normal and persister cells, co-exist. Persister cells are defined as slower growing and having reduced susceptibility to bactericidal antibiotics [19,20]. Further, bacteria can switch from one state to another. Balaban et al. proposed a mathematical model of the phenotypic switch and quantitatively analyzed the dynamics of a phenotypic heterogeneity observed in *E. coli* [21]. Since then, the model and its extensions have been used to understand the dynamics of persistence in *in vitro* culture [22–25].

*In vitro* studies have aimed to describe persister cells, which are thought to be related to the phenomenon of small colony variants (SCVs). SCVs are slow-growing phenotypic variants that form small colonies on agar plates [26]. SCVs of SA have been isolated from many clinical situations such as chronic osteomyelitis, endocarditis, and cystic fibrosis and are considered to be relevant for persistent infections [27–30]. SCVs show a poor response to antibiotics, less toxin production, and reduced susceptibility to host defenses [27,31]. Although it remains uncertain whether the formation of SCVs is a cause of SA persistence, the phenotypic heterogeneity of SA is thought to be a key feature of persistent infections. In addition, the host defense is thought to be an important factor in allowing a persistent infection. However, there is little understanding of how SA, antibiotics and host immunity work dynamically in the context of the host defense and inherent high variabilities of SA behavior.

To gain new insights into the dynamic relationship between MRSA, host immunity and antibiotic efficacy, we developed a mathematical model of persistent infection that recapitulates empirical observations from *in vitro* and *in vivo* studies. This model was designed to identify the potential key determinants that may drive persistent versus resolving MRSA bacteremia. In addition, in the simulation, we also followed a clinical practice guideline such as the timing of diagnosis of bacteremia and the treatment periods of vancomycin for RB and PB [32–34]. Interestingly, we found that distinct mechanisms are key to explaining persistence in the presence (*in vivo*) vs. absence (*in vitro*) of immune responses. *In vitro*, the degree of persistence is critically dependent on the switch rate between the normal and persister states. *In vivo*, we found that host immunity for persister cells is more important. Further, we explored pharmacological strategies that may influence persistent and relapsing bacteremia outcomes and found that a persister killer, but not a persister formation inhibitor, could provide for an effective cure the persistent bacteremia. Thus, the current modeling results point towards the importance of further experimental studies that address host-pathogen responses of persister cells, rather than antibiotic-bacterial interactions typically studied *in vitro*.

## Results

### Mathematical model for *in vitro* SA growth

**Fig 1:**
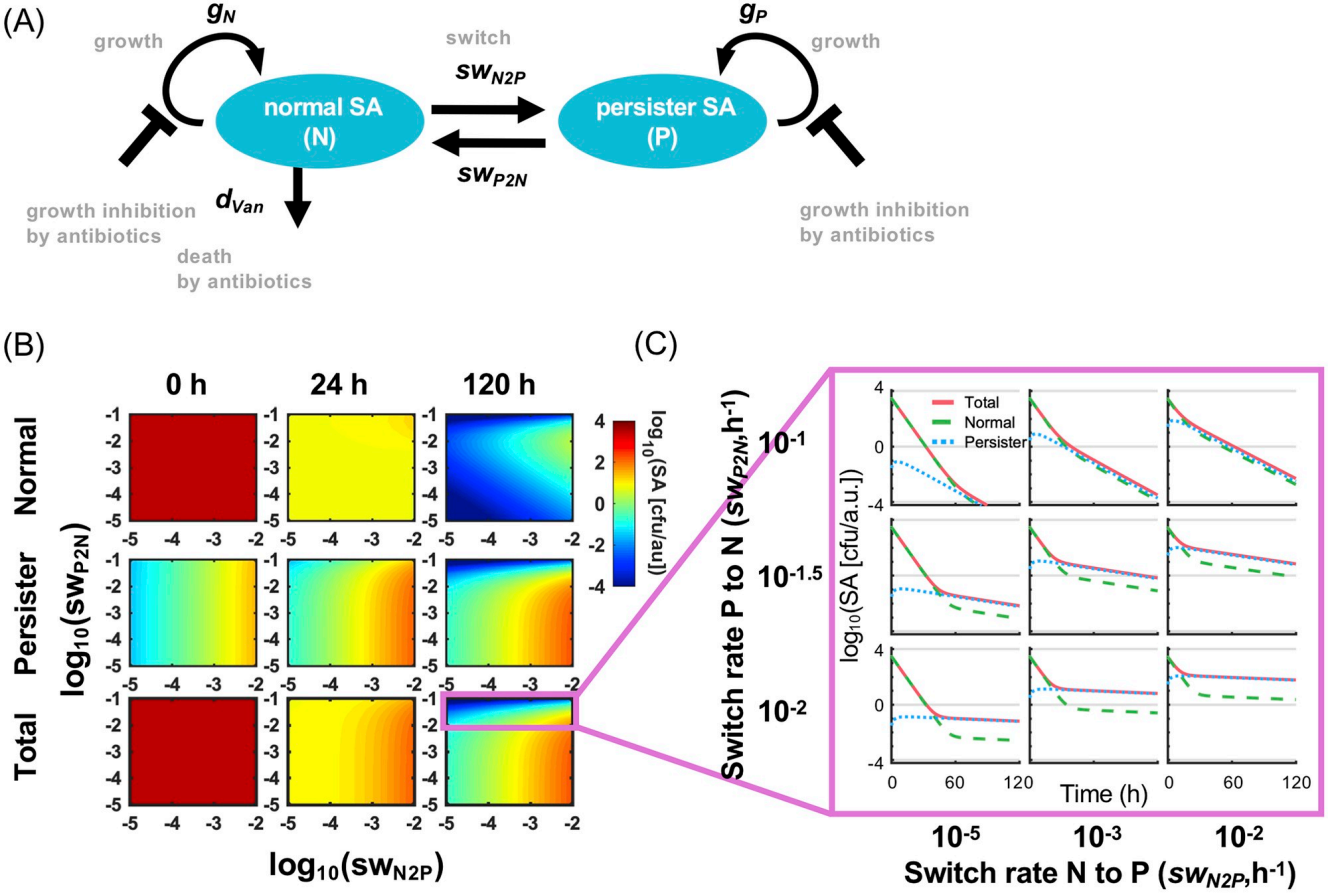
Dynamics of *in vitro* bacterial growth treated with antibiotics, vancomycin. (A) Schematic diagram of the mathematical model for *in vitro* growth. The diagram depicts the two state variables as blue ovals, normal and persister SA, whose abundances are controlled by growth rate, death rate by vancomycin (*Van*), and switch rates. The mathematical model is able to recapitulate not a phenomenon of resistance, but an *in vitro* persistence against antibiotics. (B) Heatmaps showing the number of normal, persister and total SA at 0, 24, and 120 h post-vancomycin addition as a function of switching rate *sw*_*N2P*_ and *sw*_*P2N*_. The pre-simulations were run with *N(0)* = 1 colony-forming unit per arbitrary unit (cfu/a.u.) and *P(0)* = 0 cfu/a.u. without antibiotics for 8 h, then vancomycin was added (0 h post-vancomycin addition). The parameter values used are shown in Table 1. (C) The number of total, normal and persister SA graphed as a function of time for each combination of *sw*_*N2P*_ and *sw*_*P2N*_.

To develop a mathematical model that contributes to an understanding of the mechanisms governing persistent SA bacteremia, we first developed a bacterial persistence model of SA growth *in vitro* (Fig 1) [21]. We considered that SA bacteria have two distinct phenotypic states: normal and persister cells. Normal cells are defined as those having normal growth rate and susceptibility to antibiotic and immune clearance, whereas persistent cells are defined as a minor population having relatively slower growth and whose growth may be slowed further by vancomycin but which are not directly killed by vancomycin. Our overarching hypothesis in this respect was that persistent bacteremia is attributed to the phenotypically heterogeneous bacterial populations which have differential susceptibility to antibiotics and the host immune system.

The model of *in vitro* bacterial growth consists of the following two equations:

$$\frac{dN}{dt}=-sw_{N2P}\;N+sw_{P2N\;}P+g_{N}(1-0.95\;Van)\left(1-\frac{T}{SAmax}\right)N-d_{Van}\;Van\;N$$ (1)

$$\frac{dP}{dt}=sw_{N2P}\;N-sw_{P2N}\;P+g_{P}\left(1-0.95\;Van\right)\left(1-\frac{T}{SAmax}\right)P$$ (2)

where variables *N* and *P* denote the number of normal and persister cells, respectively, in units of colony-forming unit per arbitrary unit of volume (cfu/a.u.). *T* denotes the total number of SA, a sum of *N* and *P*. Switching flux is described by the law of mass action kinetics with *sw*_*N2P*_ and *sw*_*P2N*_ being the switch rate from normal to persister cells and from persister to normal cell, respectively. Bacterial growth of normal and persister are governed by growth rate *g*_*N*_ and *g*_*P*_, respectively, with a maximum carrying capacity (*SAmax*) leading to growth saturation [24]. The saturable growth was formulated under an assumption that normal and persister cells compete for resources to grow. In *in vitro*, both normal and persister cells never reached to *SAmax* in our simulations because of the presence of vancomycin over the period of time. Both growth rates are inhibited in the presence of vancomycin (*Van*) to which a binary value of 0 or 1, absence or presence, respectively, is assigned. We assumed that vancomycin inhibits 95% of growth. Although vancomycin has potential to kill normal SA, we assumed that persister cells are not susceptible to vancomycin-mediated killing, so the death rate *d*_*Van*_ caused by vancomycin, was applied only to normal SA. The values of *g*_*N*_ and *d*_*Van*_ were taken from *in vitro* experimental data [35]. *g*_*P*_ was assumed to be 1/20 of *g*_*N*_. Table 1 summarizes the parameters used in this model.

**Table 1:** Definition and values of the parameters for *in vitro* SA growth model.

Parameter	Description	Units	Value	Range of values	Remarks
*g*_*N*_	Growth rate of normal cells	h^-1^	1.0	-	The value was taken from *in vitro* growth study [35].
*g*_*P*_	Growth rate of persister cells	h^-1^	0.05	-	Assumed that growth rate of persister was 1/20 of that of normal SA.
*sw*_*N2P*_	Switching rate from normal to persister cells	h^-1^	-	10^−5^–10^−2^	Referred to a reported value for E. coli (10^−6^–10^−3^) and SA (10^−5^–10^−3^) [24].
*sw*_*P2N*_	Switching rate from persister to normal cells	h^-1^	-	10^−5^–10^−1^	Referred to a reported value for E. coli (10^−6^–10^−1^) and SA (10^−2^–10^−1^) [24].
*d*_*Van*_	Death rate of normal cells by vancomycin	h^-1^	0.3	-	The value was taken from *in vitro* growth study [35].
*Van*	Vancomycin	-	1	-	
*SAmax*	Maximum carrying capacity	cfu/a.u.	1*10^8^	-	

Abbreviations: cfu, colony-forming unit; a.u., arbitrary unit of volume

### Simulation of the dynamics of *in vitro* SA growth treated with antibiotics

To investigate how switching rates between normal and persister SA affect the *in vitro* persistence against antibiotics (vancomycin) in the absence of host immunity, we performed a parameter scan in which the values of *sw*_*P2N*_ and *sw*_*N2P*_ were varied over at least three logs. The ranges of switching rates were determined based on the published data where *in vitro* persistence of SA or *E*. *coli* to antibiotics was evaluated mathematically [24]. Since differences in the switch rates can affect the proportion of persister cells, a pre-simulation was run for 8 h with an initial condition of *N(0)* = 1 cfu/a.u. and *P(0)* = 0 cfu/a.u., then vancomycin was added.

As seen in the heatmap (Fig 1B), varying *sw*_*P2N*_ and *sw*_*N2P*_ differentially affected the number of normal, persister and total SA. After the pre-simulation without antibiotics (0 h), normal SA reached a similar level of ~10^3.5^ cfu/a.u. in all combinations and was the major subpopulation in the culture. In contrast, the numbers of persister SA differed among the combinations, being correlated with *sw*_*N2P*_, but not with *sw*_*P2N*_. These simulation results were in concordance with the theoretical understanding [24]. After the addition of antibiotics (24 and 120 h), the number of normal SA markedly decreased. On the other hand, little change in the number of persister cells was observed for a range of *sw*_*P2N*_ between ~10^−1.5^ to 10^−5^. In this range, the number of total SA over time showed biphasic curves (Fig 1C). At the early phase, most of the total SA was killed as a result of the eradication of normal SA by vancomycin. The ensuing second phase was predominantly comprised of surviving persister cells. The changes of the second slope of total SA illustrate the comparatively slow decline of the persistent subpopulation. Thus, *sw*_*N2P*_ affected the number of persister cells after the treatment when *sw*_*P2N*_ was less than ~10^−1.5^.

On the other hand, when *sw*_*P2N*_ was greater than 10^−1.5^ h^-1^, no persistence was observed: Persister cells, as well as normal cells, were substantially killed by vancomycin at any value of *sw*_*N2P*_ (Fig 1B at 120 h and Fig 1C). These data suggested that higher values of *sw*_*P2N*_ can lead to a lack of *in vitro* bacterial persistence and may also affect the pathogenesis of persistent SA bacteremia.

### Mathematical model for *in vivo* SA growth

**Fig 2:**
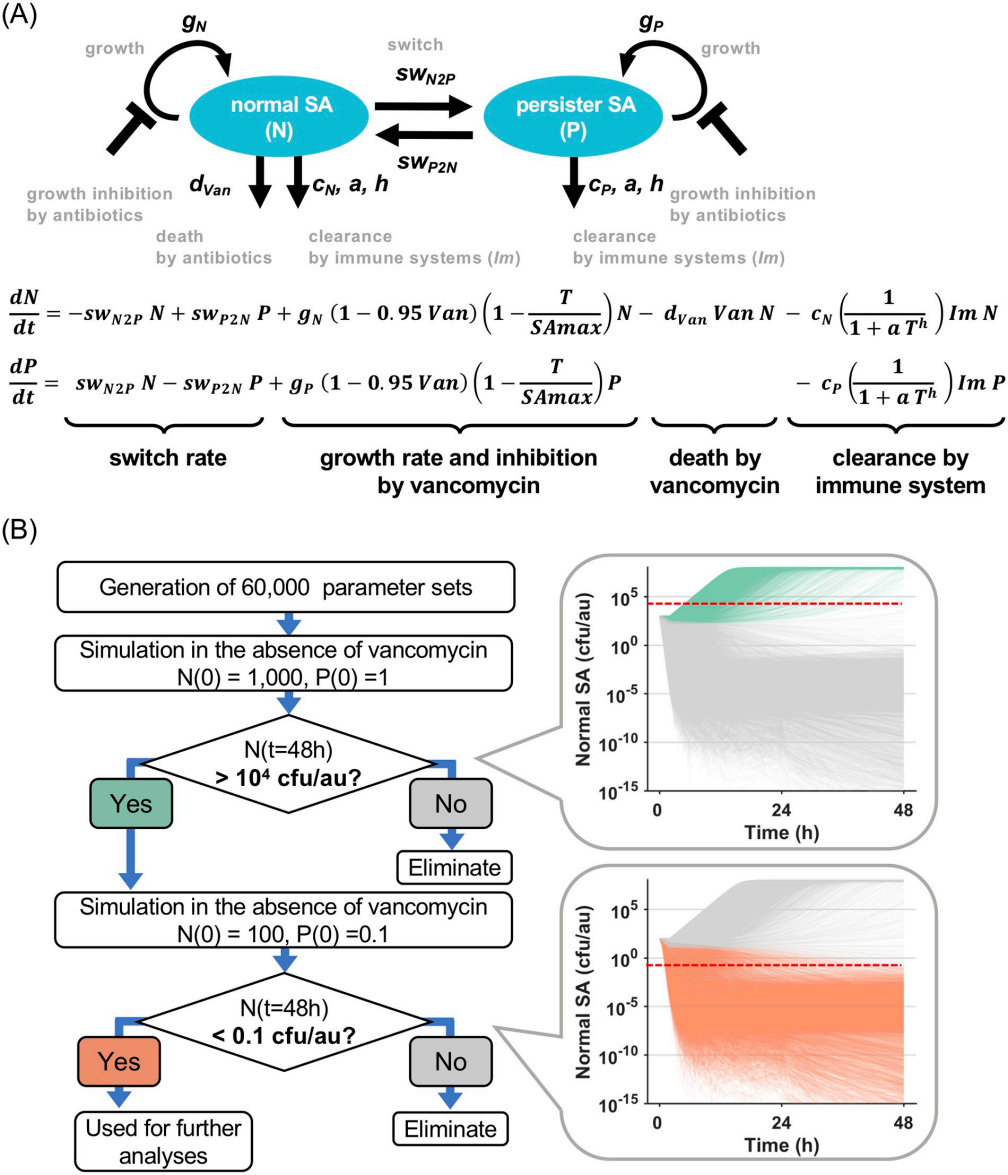
Schematic diagram of the mathematical model of *in vivo* MRSA populations and ensemble modeling. (A) The diagram depicts the two state variables as blue ovals, whose abundances are controlled by growth, death by antibiotics, clearance by the immune system (*Im*), as well as switch rates. Antibiotic treatment, vancomycin (*Van*), affects growth rates of both normal and persister and killing of the normal but not persister bacterium. The two differential equations are shown below the diagram. *Van* and *Im* are a binary value of 0 or 1 indicating absence or presence. (B) Algorithm for ensemble modeling to extract the sets of parameters (ensemble) using two criteria. All the models extracted had similar minimum inoculum doses to establish infection between around 100 to 1,000 colony-forming unit per arbitrary unit (cfu/a.u.). The parameter values randomized or fixed during the ensemble modeling are shown in Table 2.

To mathematically model *in vivo* SA growth, we expanded the *in vitro* model and added terms that describe immune system-mediated clearance (*Im*). Fig 2A shows the schematic diagram of the model and Table 2 shows the parameters and their values. The ordinary differential equations are as follows:

$$\frac{dN}{dt}={-sw}_{N2P}\;N+{sw}_{P2N}\;P+g_{N\;}(1-0.95\;Van)(1-\frac{T}{SAmax})N-d_{Van}\;Van\;N-c_{N}(\frac{1}{1+a\;T^{h}})Im\;N$$ (3)

$$\frac{dP}{dt}={sw}_{N2P}\;N-{sw}_{P2N}\;P+g_{P}(1-0.95\;Van)(1-\frac{T}{SAmax})P-c_{P}(\frac{1}{1+a\;T^{h}})Im\;P$$ (4)

where *Im* is set to 1 in all our simulations of *in viv*o infections. Both normal (*N* [cfu/a.u.]) and persister SA (*P* [cfu/a.u.]) are cleared by the immune system with clearance rates, *c*_*N*_ and *c*_*P*_, respectively. Malka et al. reported that the phagocytosis of SA by neutrophils, which has a pivotal role to eliminate SA at the early phase of infection, was saturated with a large number of SA [36]. Worlock et al. also found that to establish an *in vivo* infection, a minimum inoculum dose was necessary [37]. Hence, we describe the immune system capacity to clear bacteria as being saturable, determined by constants (*a* and *h*). The functional formulations of immune clearance used in our model are as follows:

$$c_{N}∙(\frac{1}{1+a∙T^{h}})∙Im∙N$$ (5)

$$c_{P}∙(\frac{1}{1+a∙T^{h}})∙Im∙P.$$ (6)

**Table 2:** Definition and values of the parameters for *in vivo* SA growth model.

Parameter	Description	Units	Value	Range of values	Remarks
*g*_*N*_	Growth rate of normal cells	h^-1^	1.0	-	The value was taken from *in vitro* growth study [35].
*g*_*P*_	Growth rate of persister cells	h^-1^	-	10^−3^–10^−1^	
*sw*_*N2P*_	Switching rate from normal to persister cells	h^-1^	-	10^−6^–10^−2^	Set the range based on reported values [24] and the *in vitro* model analysis.
*sw*_*P2N*_	Switching rate from persister to normal cells	h^-1^	-	10^−6^–10^−1^	Set the range based on reported values [24] and the *in vitro* model analysis.
*d*_*Van*_	Death rate of normal cells by vancomycin	h^-1^	0.3	-	The value was taken from *in vitro* growth study [35].
*c*_*N*_	Clearance rate of normal cells by immune systems	h^-1^	-	10^−0.5^–10^1^	
*c*_*P*_	Clearance rate of persister cells by immune systems	h^-1^	-	10^−4.5^–10^1^	c_P_ was randomized with keeping the ratio to c_N_ between 10^−4^ and 10^0^ by using the following equations: c_P_ = f x c_N_ where f was log-uniformly randomized between 10^−4^ and 10^0^.
*a*	Saturable effect of the clearance against total number of SA		-	10^−1.5^–10^0.5^	
*h*	Hill coefficient for total number SA	-	-	0.2–2.0	
*Im*	Immune systems	-	0 or 1	-	
*Van*	Vancomycin	-	1	-	
*T(t)*	The number of total SA at time t	cfu/a.u.	*N(t)+P(t)*	-	
*SAmax*	Maximum carrying capacity	cfu/a.u.	1*10^8^	-	

In the ensemble modeling, the values of *g*_*P*_, *sw*_*P2N*_, *sw*_*N2P*_, *c*_*N*_, *c*_*P*_, and *a* were randomized log-uniformly and the values of *h* were randomized uniformly in the range shown in Range of values. Abbreviations: cfu, colony-forming unit; a.u., arbitrary unit of volume or weight.

These formulations are based on the following mathematical equation to represent *in vitro* saturable SA clearance by neutrophils [36]:

$$kill\;rate=\frac{\alpha ∙Neu∙Bac(t)}{1+\gamma ∙Bac(t)+\eta ∙Neu}$$ (7)

where *Neu* and *Bac(t)* represent the number of neutrophils and bacteria, respectively. α is the neutrophils’ bacterial killing rate at low concentrations, and γ and η control the saturation in the killing rate as Bac and Neu are increased, respectively. The equation can be also expressed as follows:

$$kill\;rate=\frac{\alpha }{1+\eta ∙Neu}∙\frac{1}{1+\frac{\gamma }{1+\eta ∙Neu}∙Bac(t)}∙Neu∙Bac(t).$$ (8)

*In vivo*, not only neutrophils but also macrophages play a role in clearing SA, thus *Neu* was generalized as clearance by immune cells (*Im*) in our model. Further, under the assumption that the number of these innate immune cells is constant, we derive the equation used in our model:

$$kill\;rate=c_{N}∙\frac{1}{1+a∙Bac(t)}∙Im∙Bac(t)$$ (9)

$$a=\frac{\gamma }{1+\eta ∙Im}$$ (10)

$$c_{N}=\frac{\alpha }{1+\eta ∙Im}.$$ (11)

In our formulation, we also introduce the Hill function to account for how the clearance rate is a function of the number of bacteria.

**Fig 3:**
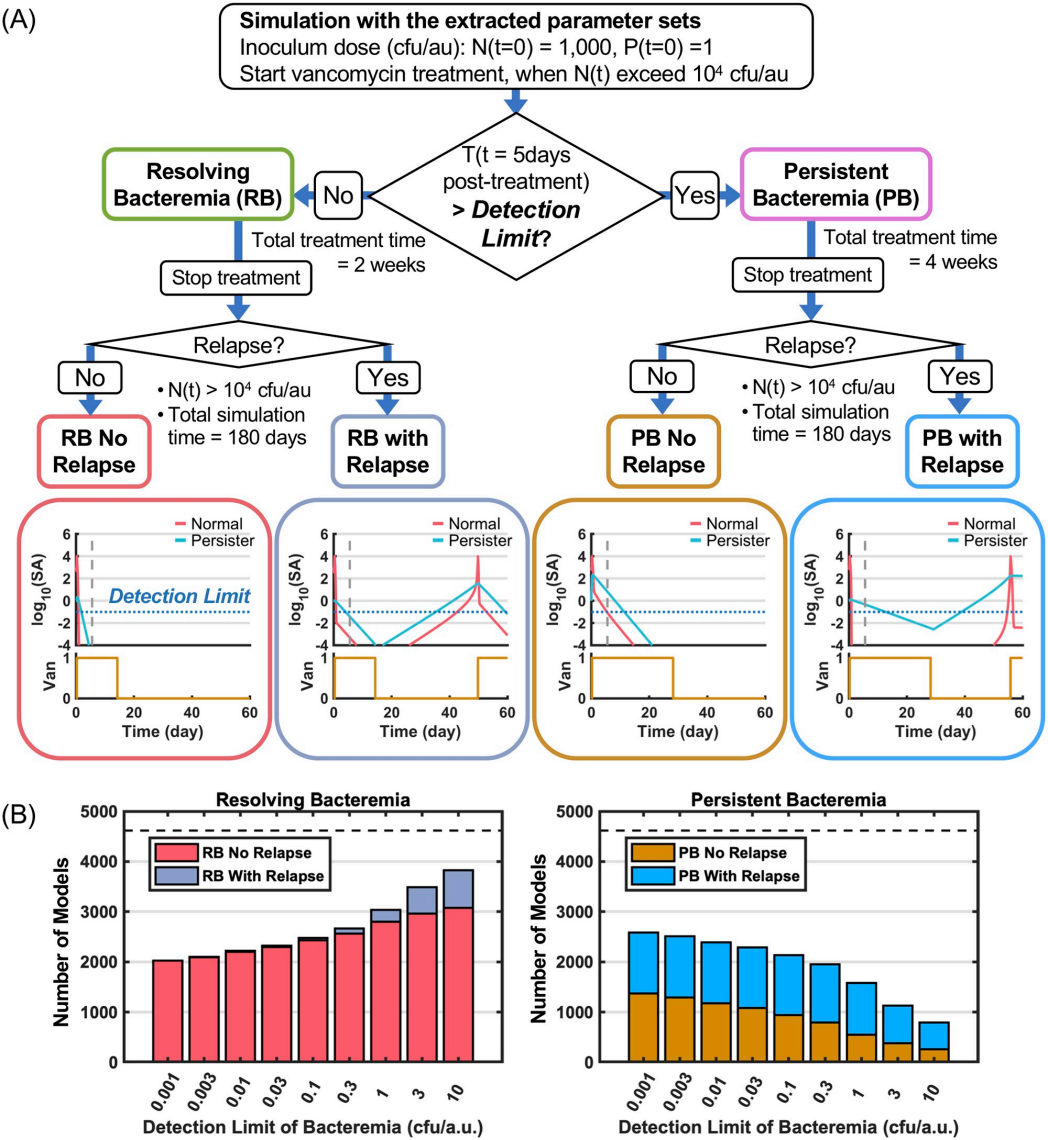
Simulation of the models with vancomycin therapy. (A) The selected parameter sets were used for simulation with vancomycin treatment and classified into resolving (RB) and persistent bacteremia (PB) as described in the algorithm. Vancomycin was administered when normal SA (*N*) exceeded 10^4^ cfu/a.u. The models were judged as PB when the number of total SA (*T*) at 5 days post-treatment exceeded a detection limit under an assumption where positive bacteremia is observed when the number of SA at infection foci exceed a certain number (see text in details). The detection limit of bacteremia is set as 0.1 cfu/a.u. in this study under an assumption where relapse bacteremia is less in RB (see Fig 3B). The relapse bacteremia was determined when vancomycin was re-administered triggered by regrowth of SA after 1^st^ vancomycin treatment. Four cases of typical simulations, RB no relapse, RB with relapse, PB no relapse and PB with relapse, are shown in the bottom. (B) Bar plots show the number of models with or without relapse for RB (left) and PB (right) over a range of detection limit from 0.001 to 10 cfu/a.u. A dotted line indicates the total number of models.

In general, bacterial counts in a patient’s blood is not high, because bacteria in the blood are cleared rapidly within several minutes to 1 hour [38]. Hence, in clinical practice, bacteremia is diagnosed in a qualitative manner, positive or negative, by culturing the patient’s blood on the plate. Hence, due to the lack of quantitative data availability the *in vivo* model does not include an explicit blood compartment. Instead, we assumed that when the number of SA in infection sites exceeded a threshold number, then, MRSA would be detected in the patient’s blood, since SA detected in blood derives from ongoing infection foci. We explored a range of values for the detection limit of bacteremia and showed how our conclusions remain robust to them (Fig 3B, S1 Fig).

To simulate disease using this model we identified two challenges: inherent uncertainties about the quantitative behavior of bacteria and the immune system, and about the appropriate inoculum size. To address both, we performed ensemble modeling to select parameter sets (ensemble) which had resulted in informative disease courses.

### Ensemble modeling for *in vivo* SA growth

The goal of ensemble modeling is to generate a set of kinetic models (ensemble) where each model is described by different sets of kinetic parameters but the same mathematical structure, and outputs match specified target behavior, as for example defined by experimental or clinical data [39]. An advantage of this approach is that it enables accounting for uncertainties related to kinetic parameters which are unknown or difficult to determine experimentally, while the emergent properties of the model must satisfy a set of known biological phenomena. Thus, ensemble modeling is an often used approach in systems biology, especially in metabolic modeling [40]. Here, we applied the ensemble modeling approach to *in vivo* SA growth model where inherent uncertainties exist about bacterial growth behavior and the effectiveness of the host immune system and selected parameter sets which led to establishing an infection.

The workflow of the ensemble modeling is schematized in Fig 2B. First, we generated 60,000 sets of parameters for *g*_*P*_, *sw*_*P2N*_, *sw*_*N2P*_, *c*_*N*_, *c*_*P*_, *a*, and *h*. On the basis of the definition of persister cells, the growth rate of persister SA, *g*_*P*_, was randomized to generate values less than those for normal SA. *sw*_*P2N*_, *sw*_*N2P*_ were randomized in ranges shown in Table 2 based on the reported values. *c*_*N*_ and *c*_*P*_ were randomized so that *c*_*P*_ was always less than or equal value of *c*_*N*_. Details are described in Table 2. Among the model parameters, *g*_*N*_, *d*_*Van*_, and *SAmax* were fixed in the ensemble modeling. The value of *g*_*N*_ was taken from *in vitro* growth study [35]. Pharmacodynamics effects of vancomycin, *d*_*Van*_ and the growth inhibitory effect (0.95), were fixed because no difference in vancomycin concentration was observed between RB and PB patients [41,42]. Further, the value of *d*_*Van*_ was taken from the *in vitro* study and used for the ensemble modeling [35], because the concentration of vancomycin evaluated *in vitro* appears similar to that in patients, based on the following observations. The death rate of vancomycin was estimated *in vitro* at a concentration of 10-fold of MIC. In patients, AUC (24h) / MIC was reported to be 199–426 [42], indicating, on average, vancomycin plasma concentration was 8.3–17.8 fold higher than MIC. Since plasma protein binding of vancomycin was reported to be about 40% [43], we conclude that the clinical vancomycin concentration as an unbound form was around 5–10 fold higher than MIC, which is comparable to *in vitro* concentration used to estimate the death rate induced by vancomycin.

After generating the parameter sets, we selected them which for a given inoculum dose range would establish an infection (Fig 2B). We hypothesized that the minimum inoculum dose was between around 100 and 1,000 cfu/a.u and set two criteria to extract parameter sets: an inoculum dose of ~1,000 cfu/a.u. will establish a productive infection in the absence of antibiotic treatment, and an inoculum dose of ~100 cfu/a.u. will not (Fig 2B). In the 1^st^ regime, we conducted simulations for 48 h at an inoculum dose of 1,000 cfu/a.u. of normal SA and 1 cfu/a.u. of persister SA in the absence of vancomycin (*Van* = 0); Since persister cells were a minor population of SA, we set the proportion of persister cells to 0.1% of normal SA. In the *in vivo* simulation, a 2 h of lag-time was introduced so that bacterial growth begins at 2 h post-inoculation. Then we performed simulations with the parameter sets, evaluated the number of normal SA at 48 h and extracted parameter sets which exceed 1x10^4^ cfu/a.u. for the subsequent criteria. In the 2^nd^ regime, we ran simulations at an inoculum dose of 100 cfu/a.u. and 0.1 cfu/a.u. of normal and persister cells, respectively, using the extracted parameter sets from the previous step, then selected parameter sets which met criteria in which the number of normal SA at 48 h was less than 0.1 cfu/a.u.

Of 60,000 initial parameter sets, we obtained 4,614 of parameter sets whose minimum inoculum dose to establish infection was between around 100 and 1,000 cfu/a.u, and used them for further simulations. The inoculum dose chosen was 1,000 cfu/a.u. of normal SA and 1 cfu/a.u. of persister SA (0.1% of normal SA), unless otherwise noted, at which all the parameter sets established a productive infection in the absence of vancomycin.

### Simulation with the extracted parameter sets under vancomycin therapy and algorithm to classify them into RB and PB

With the goal of classifying the selected parameter sets into RB or PB, we performed the *in vivo* SA growth simulation with each of the parameter sets under standard vancomycin therapy (Fig 3A). In clinical practice, PB is defined by positive blood cultures on 3–7 days post-therapy [1,6,7]. In our simulation, we followed a similar diagnostic process by introducing a detection limit of bacteremia to the number of total SA (*T*) at 5 days post-treatment by vancomycin (Fig 3A). Two central assumptions were that SA detected in blood derive from ongoing infective foci, and when the number of SA in infection sites exceeds a certain number (detection limit of bacteremia), then, MRSA can be detected in the patient’s blood. First, we explain the algorithm to classify the models, then we describe how we set the detection limit of bacteremia.

In the *in vivo* simulation under vancomycin therapy (Fig 3A), the simulations were initiated at a dose of 1,000 cfu/a.u. of normal SA and 1 cfu/a.u. of persister SA without vancomycin, with bacterial growth starting at 2h post-inoculation. When normal SA reached the threshold of 1x10^4^ cfu/a.u., vancomycin was administered (*Van* = 1). As the trigger for the onset of treatment, we employed not total SA but normal SA, because persister cells are potentially less virulent [28]. Hence, we assumed that persister SAs do not provoke symptoms of infections, such as fever, and are therefore not a trigger of treatment. At 5 days post-treatment, the models were judged as PB if the number of total SA (*T*) exceeded a detection limit, and judged as RB if the number of total SA were below the detection limit. Based on clinical guidelines for MRSA bacteremia [32–34], different treatment periods of vancomycin were applied for RB and PB: When a model was classified at 5 days of treatment as RB, treatment time was terminated at 2 weeks, but when it was classified as PB, the treatment time was extended to 4 weeks.

Further, RB and PB could be sub-classified based on whether they relapsed or not (Fig 3A, bottom); In case of non-relapsing case, vancomycin therapy (in conjunction with the immune system) effectively eradicated both normal and persister SA. In contrast, in cases of relapse, vancomycin eradicated normal SA, but treatment is not sufficient to eradicate persister SA. The surviving persister cells are able to proliferate and switch to normal SA, accelerating bacterial growth until they reach a level of clinical presentation (>10^4^ cfu/a.u.) that then trigger a subsequent round of treatment. In this study, relapse bacteremia was defined as at least two cycles of vancomycin therapy over the 180 days observation period. Here, we found that there were 4 types of simulations: RB no relapse, RB with relapse, PB no relapse, and PB with relapse; typical simulation results are shown in the bottom of Fig 3A.

Next, to set an appropriate value for the detection limit of bacteremia, we explored the value under the assumption that relapse of bacteremia is rarely observed in the RB scenario. We found that when the detection limit is set to 0.3 cfu/a.u. or lower, very few models that satisfy the RB condition would lead to relapsing bacteremia (Fig 3B). In this range, interestingly, the number of models that satisfied the RB condition did not change very much (from approximately 2,500 to 2,000) over a >300-fold range of the detection limit. For further study, we selected 0.1 cfu/a.u. as a detection limit of bacteremia.

By applying the detection limit of 0.1 cfu/a.u. to the algorithm shown in Fig 3A, we found that of 4614 models, 2,479 models (53.7% of total) and 2,135 models (46.3% of total) were classified into RB and PB, respectively. Further, of the 2,479 RB models, 2,427 models led to clearance (RB no relapse, 52.6% of total) and 52 to relapsing bacteremia (RB with relapse, 1.1% of total). In contrast, of the 2,135 PB models, 940 led to clearance (PB no relapse, 20.4% of total), and 1,195 led to relapsing bacteremia (PB with relapse, 25.9% of total).

### Comparison of the parameter sets between RB and PB

**Fig 4:**
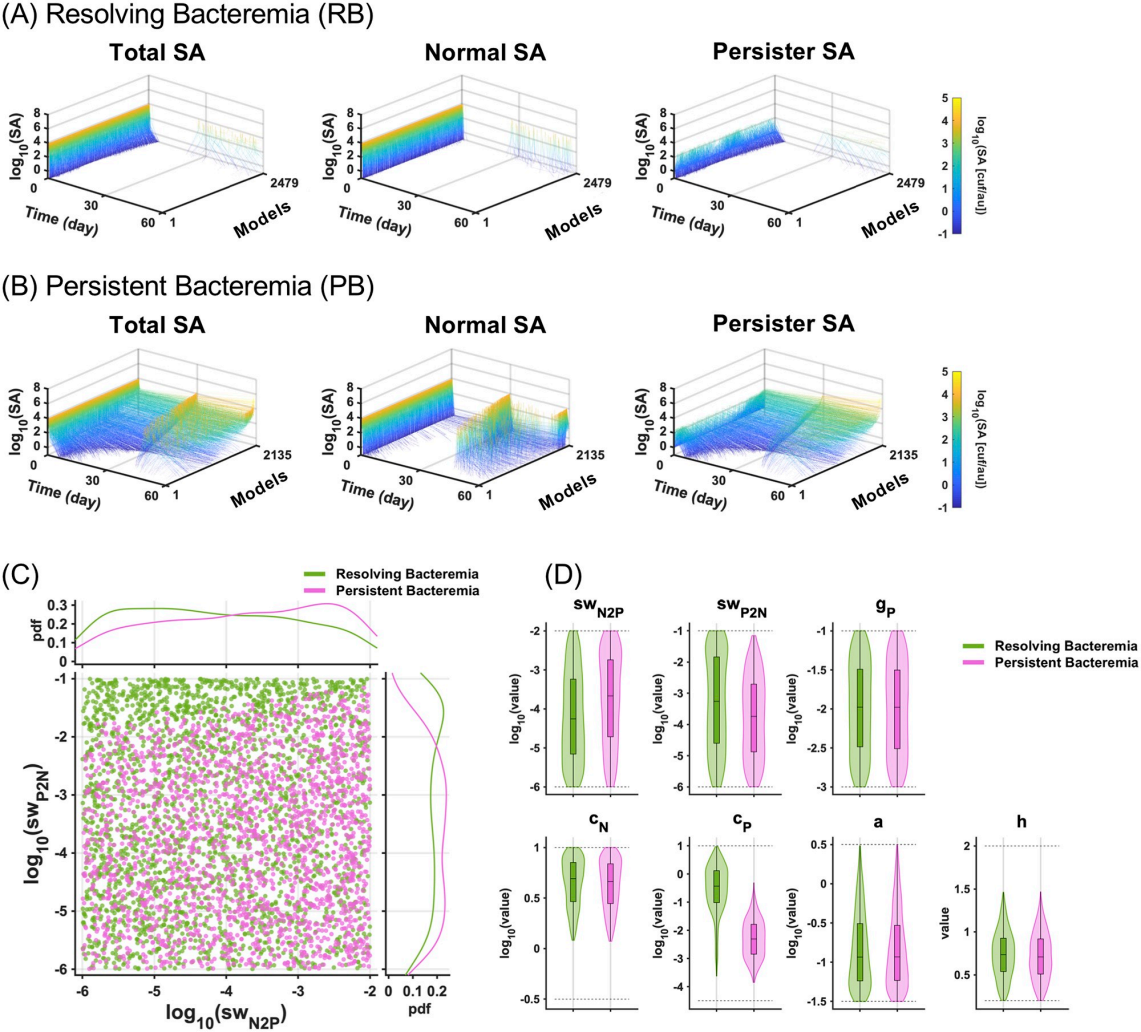
Comparison of models between resolving and persistent bacteremia. (A, B) Line plots of the number of total, normal, and persister SA over time (0–60 days) for resolving (RB) and persistent bacteremia (PB). The detection limit of bacteremia was set as 0.1 cfu/a.u. and 2,479 and 2,135 models were classified into RB and PB, respectively. In RB, 52 of models showed relapsing bacteremia and they were also included in the analysis. (C) Two-dimensional scatter plot of switching rates. (D) Violin plots and box plots of the parameter distributions of RB and PB. Dotted lines indicate the range of randomized parameter values.

To examine the cases defined by these models in further detail we graphed the timecourses of the simulations. For the 2,479 models classified as RB, bacterial numbers indeed dropped rapidly upon vancomycin administration, and only very few models showed relapse (Fig 4A). In contrast, for 2,135 models classified as PB, bacterial numbers dropped more slowly, and only partially in the majority of outcomes (Fig 4B). The residual bacteria were due to a substantial persister SA population that is less susceptible to vancomycin treatment in the *in vivo* model. Indeed, normal SA was largely eradicated by treatment, but only recurred after treatment cessation, presumably driven by the substantial persister population.

Because switch rates between persister and normal SA were key determinants of persistence *in vitro* (Fig 1B and 1C), we examined whether these parameters were also important for determining *in vivo* persistence. We plotted *sw*_*P2N*_ and *sw*_*N2P*_ values for above-defined *in vivo* RB and PB models (Fig 4C). Remarkably, we saw no effect when the switch rate from persister to normal cells (*sw*_*P2N*_) or the converse rate (normal to persister, *sw*_*N2P*_) were altered, except at very high values of *sw*_*P2N*_, akin to what we observed in *in vitro* simulations where a lack of persistence was observed when *sw*_*P2N*_ was greater than around 10^−1.5^ h^-1^ (Fig 1B and 1C). These data implied that the switch rates were not strong determinants of PB and RB *in vivo*.

Examining all parameter values in the selected models, we found that only the *c*_*P*_ parameter, which determines the susceptibility of persister SA to immune clearance, demonstrated clearly different distributions between RB and PB outcomes (Fig 4D). This implied that *c*_*P*_ might be a key determinant of PB vs. RB. Further, we found that *c*_*N*_, *c*_*P*_, *a*, and *h* exhibited non-uniform distributions and narrower ranges than the ranges we randomized. These data indicated that these parameters, which are related to clearance of SA by immune systems, were sensitive to the minimum inoculum dose used as criteria in the ensemble modeling. Thus, although the parameter ranges we considered may be narrower than the ranges of other parameters (Table 2), they are sufficient to fully explore the emergent properties of the model within the criteria applied in the ensemble modeling approach.

### Key determinants to distinguish between RB and PB

**Fig 5:**
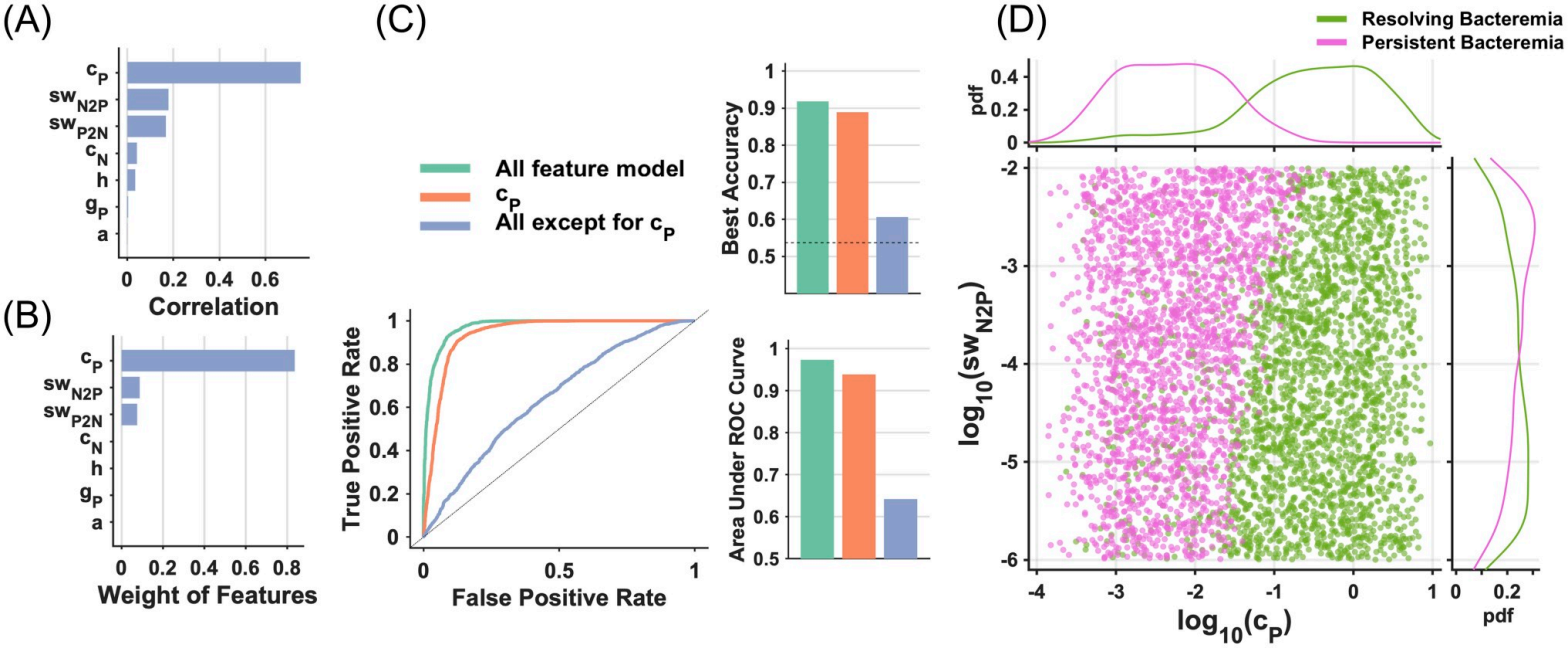
Key determinants to distinguish resolving and persistent bacteremia. (A) Pearson correlation coefficients were calculated between each feature and the binary response variable (0: RB, 1: PB). The absolute values of the coefficients are shown. (B) Weights calculated by QPFS methods are shown. (C) The sole ranked feature by QPFS, *c*_*P*_, was used to build a logistic regression model. For the comparison, all parameter and all parameter except for *c*_*P*_ were applied to the classification model. Best accuracy, ROC curve, and area under ROC curve are graphed for each model. (D) Two-dimensional scatter plot for *c*_*P*_ and *sw*_*N2P*_, which showed 2^nd^ high correlation with target response (Fig5 A) with probability density for RB and PB.

To identify the key determinants of RB vs. PB outcomes in an unbiased manner, we pursued a machine learning approach. First, we applied the quadratic programming feature selection (QPFS) method [44], which assigns relative influence weights to given features in a manner that minimizes redundancy while maximizing relevance (see Methods). Here, relevance was calculated as Pearson correlation coefficients between each feature and the binary target response (Fig 5A). Graphing the weights of each parameter obtained by QPFS (Fig 5B) demonstrated that *c*_*P*_ was selected as a sole feature for the subsequent classification.

To evaluate the ability of the selected features to discern RB and PB, we used logistic regression to model the log-odds ratio of the probability of PB as a linear combination of the features. The logistic regression models for each combination of the features were evaluated by the Receiver-Operator Characteristic (ROC) curve and the area under the ROC curve (AUC, Fig 5C). Compared with the AUC of a model that used all features, the model consisting only of *c*_*P*_ showed an equivalent performance. This result indicates that the sole feature of *c*_*P*_ identified by QPFS is sufficient to construct an accurate classification model with the best accuracy of 93.2%, which was comparable to the model encompassing all features (97.0%). A two-dimensional plot of *c*_*P*_ and *sw*_*N2P*_, which showed the 2^nd^ highest correlation with target response (Fig 5A), indicates that *c*_*P*_ is clearly able to distinguish between RB and PB (Fig 5D). Thus, *c*_*P*_ was identified as a sole and key determinant differentiating RB from PB outcomes in this *in silico* model.

### Key determinants to distinguish resolving, persistent and relapsing bacteremia

**Fig 6:**
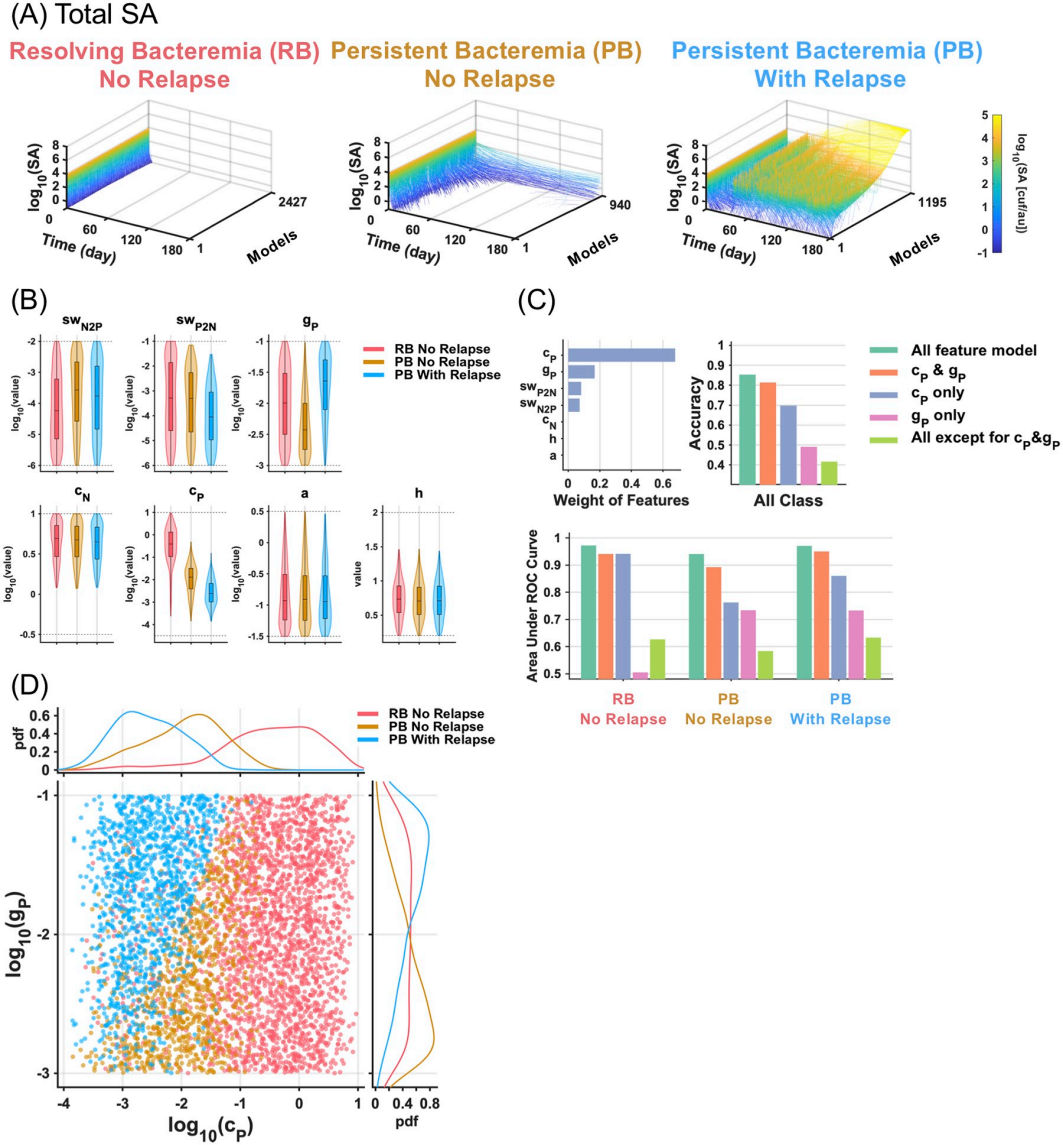
Key determinants to distinguish resolving, persistent and relapsing bacteremia. (A) Line plots of the number of total SA over time (0–180 days) for resolving bacteremia (RB) without relapse, persistent bacteremia (PB) without and with relapse bacteremia. (B) Violin plots and box plots of the parameter distributions. Dotted lines indicate the range of randomized parameter values. (C) Weights calculated by QPFS methods are shown. The ranked features by QPFS, *c*_*P*_ and *g*_*P*_ were used to build a multinomial logistic regression model. For the comparison, all parameter and all parameter except for *c*_*P*_ and *g*_*P*_ were applied to the classification model. The overall accuracy and area under ROC curve for each class are graphed. In the classification, an equal number of datasets in each class were used by reducing the number of data in major classes, RB without relapse and PB with relapse, to the same number of minor class, 940 of PB without relapse. (D) Two-dimensional scatter plot for *c*_*P*_ and *g*_*P*_ with probability density of each value.

Having identified the determinants of resolving vs persistent bacteremia (RB vs PB), we next explored the key determinants of relapsing bacteremia, which resulted from roughly half the models that showed persistence at 5 days of treatment. As described (Fig 3A), almost all (2,427 vs 52) of the RB models did not result in relapse, whereas 1,195 vs 940 PB models did result in relapsing bacteremia (Fig 6A). The timecourse data shows that in the large majority of relapsing outcomes, substantial bacteremia occurs shortly after the 4 weeks treatment is terminated, and typically by 60 days of the timecourse. Even following repeated cycles of vancomycin treatment, the bacteremia relapses without fail. Examining the parameter distributions for the three distinct scenarios revealed that PB with relapsing bacteremia is associated with different parameter distributions in *c*_*P*_ and *g*_*P*_ than those of RB or PB that do not relapse (Fig 6B).

To identify the key determinants to distinguish relapsing bacteremia, we first performed feature selection by QPFS as before, then applied the key features to build a multinomial logistic regression model, an extension of the logistic regression for a multi-class problem. By QPFS, not only *c*_*P*_ but also *g*_*P*_, the growth rate of persister cells, were ranked as relevant features to classify resolving, persistence and relapsing outcomes (Fig 6C, upper left). These two features were able to show comparable overall accuracy to the all-feature model (Fig 6C, upper right). The AUC for each class indicated that for the class of RB without relapse, an only-*c*_*P*_ model was able to show comparable performance to an all-feature model, whereas both features *c*_*P*_ and *g*_*P*_ were necessary to distinguish the classes of PB with or without relapse (Fig 6C, bottom). A two-dimensional plot of *c*_*P*_ and *g*_*P*_ is clearly able to discern these three classes (Fig 6D), indicating that *c*_*P*_ and *g*_*P*_ were important factors that discriminate relapsing from non-relapsing PB. This finding suggests that while immune clearance mechanisms targeting persister cells critically determine resolving and persistent bacteremia, the growth rate of persister cells further determines whether persistent bacteremia is relapsing or not.

### Pharmacological strategies for persistent and relapsing bacteremia

**Fig 7:**
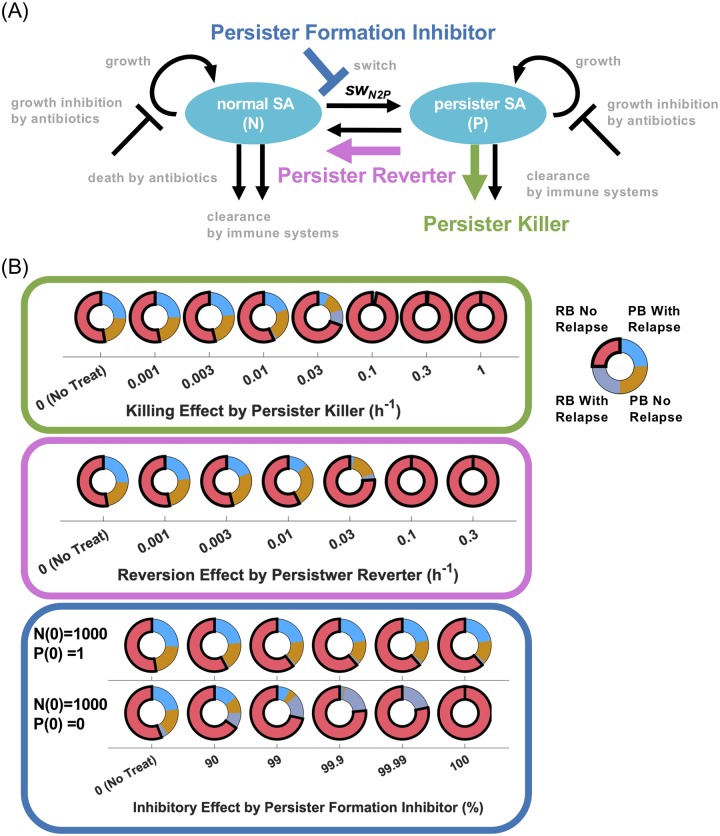
Pharmacological strategies for persistent and relapsing bacteremia. (A) Three possible pharmacological strategies, persister killer, persister reverter and persister formation inhibitor, are shown on the schematic diagram of *in vivo* MRSA model. (B) Doughnut charts show the number of models for resolving bacteremia (RB) without relapse, RB with relapse, persistent bacteremia (PB) without relapse, and PB with relapse by the treatment of three drug candidates in the presence of vancomycin. The pharmacological strengths of persister killer and persister reverter are expressed as a kinetic rate (h^-1^). During the simulation, persister killer and persister reverter were administrated in conjunction with vancomycin. Persister formation inhibitor was treated from the beginning of the simulation with different initial numbers of persister cells, 1 or 0 cfu/a.u.

Having identified that the key determinants of SA pathogenesis were related to persister cells and their interactions with the host, we investigated three possible types of drug strategies affecting persister bacteria. We termed these “persister killer”, “persister reverter” and “persister formation inhibitor” (Fig 7A). The pharmacological strength of persister killer and persister reverter regimens were expressed as kinetic rates and simulated by mass action kinetics. During the simulation, persister killer and reverter regimens were administrated in conjunction with vancomycin which was administered for 2 or 4 weeks for RB and PB, respectively, when normal SA reached to 1x10^4^ cfu/a.u, the defined trigger for the onset of treatment. Persister killer regimens were able to achieve complete remission of PB at 0.1 h^-1^, which is one-third of the killing rate of normal SA by vancomycin (Fig 7B, top). The reverter regimen could also clear the PB by 0.1 h^-1^ (Fig 7B, middle). Because this rate is comparable to the reported highest value of switch rates from persister to normal for SA and *E.coli* [24], the reverter regimen may achieve the pharmacological effect if it can maximally activate the switch rate from persister to normal. The persister formation inhibitor inhibits the switch from normal to persister cells and its strength was expressed as a percent of inhibition. Unlike the killer and reverter regimens, the inhibitor was administered from the beginning of the simulation to investigate the maximum pharmacological effects with different initial numbers of persister cells, 1 or 0 cfu/a.u. When the initial number of persister cells is 1 cfu/a.u., which is the same initial condition applied for other analysis, the persister formation inhibitor could not achieve complete remission, even in the 100% inhibition of the switch process (Fig 7B, bottom). On the other hand, when the initial number of persister cells was set to 0, complete remission was achieved by 100% inhibition, but not by 99.99% prevention. Hence, persister formation inhibitor regimens may not have the potential to cure PB. Thus, these data indicated that persister killer may be the most promising and robust therapeutic strategy to resolve PB. Note that, in this analysis, complete remission achieved by the hypothetical regimens does not suggest a complete response in all patients. However, it does indicate that persister killer is a robust pharmacological strategy under any condition (parameter values) because the parameter sets were generated by merely randomizing values within plausible ranges without knowledge of the actual parameter distributions.

## Discussion

Persistent infection by MRSA, in which antibiotics fail to clear the infection, can be a cause of life-threatening persistent bacteremia. However, MRSA isolates from PB cases remain susceptible to antibiotics *in vitro* [1]. As a plausible mechanism of *in vivo* persistent infection, a phenotypic heterogeneity within the bacterial population with co-existing normal and persister cells has been considered [21]. However, little is known about the dynamic relationships between SA, antibiotics and host immunity to explain *in vivo* persistent infections. Thus, the factors or mechanisms that determine whether antibiotic treatment results in RB or PB have been elusive. An understanding of the determinants governing PB which is invoked uniquely in the context of *in vivo* infection will facilitate the identification of biomarkers to predict the outcomes and the development of novel therapeutic interventions to avoid persistent infection.

In this study, we addressed the dynamic interactions between SA, vancomycin treatment and host immunity by developing a mathematical model consisting of two different bacterial phenotypes—normal and persister cells—that can switch from one to another. Persister cells were defined as slow growing and lacking susceptibility to direct killing by antibiotics. Because inherent uncertainties exist about bacterial growth behavior and the effectiveness of the host immune system, we applied an ensemble modeling approach to identify a single critical parameter that discerns RB and PB: the clearance rate of persister cells by the immune system (*c*_*P*_). The results clearly indicated that the survival of persister cells played a critical role in establishing PB (Fig 5D). To our surprise, unlike *in vitro* persistence (Fig 1), the switch rates between normal and persister cells were not critical to establishing PB (Fig 4C). In *E*. *coli*, the formation of persister cells has been well-investigated and found to be linked to toxin-antitoxin modules and/or to ATP-dependent mechanisms [19,45]. In SA, negligible effects of toxin-antitoxin modules are observed and ATP levels (e.g. energetics or formation of small colony variants) may have roles in persistence [19,46]. While important, these prior investigations do not address how persistent infections emerge, or their natural history *in vivo*. Thus, studies investigating the intersection among persister cells, host immunity and antibiotic therapy will give us new insights for understanding *in vivo* persistent infections.

Intracellular SA may be a promising explanation for the persister cell properties that cause persistent infections *in vivo*, as exploitation of the intracellular compartment can facilitate immune evasion and reduced susceptibility to antibiotics. Until recently, SA was thought to be an exclusively extracellular pathogen [26]. However, there is now accumulating evidence that SA is able to survive within host cells, both professional and non-professional phagocytes. Kubica et al. reported that SA remained within macrophages for 5 days post-infection [47]. Here, phagocytosed SA localized in acidic subcellular compartments and were able to replicate inside the cells before lysing the cells to exit [48]. The cycle of lysis and re-uptake may maintain a pool of viable SA over time [49]. In addition, SA is also able to live and proliferate inside non-professional phagocytes, such as endothelial cells and epithelial cells [49,50], after entering these cells *via* specific receptor-ligand interaction, which facilitates adhesion and can prompt internalization [51]. Specifically, phenotypic variants (e.g. SCVs) are also able to reside in the intracellular compartments of phagocytic and non-phagocytic cells [52,53]. Intracellular SCVs have been recovered from patients with chronic osteomyelitis and cystic fibrosis years after the initial infection [28,30]. Because methods to detect SCVs experimentally still have difficulties due to the slow growth rate and the reversion to normal SA, it is unclear whether SCVs are an exclusive phenotype of intracellular SA.

Although our mathematical model did not contain intracellular SA explicitly, intracellular SA appears to have the same characteristics as the persister cells in our model: relatively slow growth rate, and reduced susceptibility to antibiotics and immune clearance [26,49,50,52]. Hence, we hypothesize that persister and normal cells represent intracellular and extracellular SA *in vivo*, respectively. Thus, our conclusion that the clearance of persister cells by the immune system (*c*_*P*_) was identified as a key determinant of PB vs. RB outcomes, may be directly relevant to the phenomenon of intracellular persistence. In this respect, key determinants may be the interaction between intracellular SA and infected host cells: how infected cells kill intracellular SA and how intracellular SA evade this killing. Indeed, these processes may be affected by bacterial and/or host factors, both genetic and non-genetic. On the other hand, our work suggests that the switching processes between normal and persister cells are not critical to persistence. In this respect, the entering/exiting process whereby extracellular SA and intracellular SA undergo phenotypic switching may not be as important to the establishment of a persistent infection *in vivo*.

Our modeling predicted that a persister killer strategy is the most promising and robust drug strategy to cure PB (Fig 7). Recently, as an agent to target intracellular SA, antibody-antibiotics conjugate (AAC) has been applied to the *in vivo* intracellular MRSA model, showing good efficacy compared to vancomycin [54]. Kim et al. also reported a new class of synthetic retinoid antibiotics which kill MRSA persister strains [55]. Being able to kill the persister cell directly, therapeutics such as these may hold promise to resolve the persistent infection. In addition to directly killing persister cells, inhibiting the evasion mechanism of persister cells and enhancing host defense activity against persister cells may also improve outcomes. On the other hand, we found that preventing persister emergence may not be efficacious. For example, our modeling results indicate that inhibiting the entry process *via* blocking receptor-ligand interactions via antibodies may not be as promising an anti-infective strategy against intracellular persistence [56,57].

For the purposes of identifying key parameters to distinguish PB and RB, we employed machine learning approaches, instead of global parameter sensitivity analyses, such as partial rank correlation coefficient (PRCC), which explore the impact of each parameter value [58]. In PRCC, multiple parameter sets are generated by randomizing the values, then simulations are performed with the parameter sets. To find the key sensitive parameters, correlations between parameter values and the simulated data of interest are evaluated. However, in our analysis, global parameter sensitivity analyses are not appropriate, because certain parameter sets may show no bacterial growth *in vivo* even in the absence of vancomycin and may thus lead to the misleading conclusion that such parameter is a key determinant of RB. Further, while parameter sensitivity analysis is a powerful way to evaluate the impact of each parameter, it is difficult to address the effects of multiple parameters, as observed in Fig 6. Thus, the combination of simulations with the parameter sets and machine learning classification may be a powerful approach to identify the key parameter combinations in complex dynamical systems and is applicable to identify biomarkers of pathogenesis and treatment efficacy.

In line with expectations, in our model of *in vitro* MRSA growth, the fraction of persister cells was a function of the switch rates, especially the switch rate from normal to persister cells (*sw*_*N2P*_) as shown in Fig 1C. The initial fraction of persister cells at *sw*_*N2P*_ of 10^−5^, 10^−3^, 10^−2^ h^-1^ was 9.0–0.8 x 10^−4^, 8.9–9.7 x 10^−2^, and 8.4–9.2 x 10^−1^%, respectively, when *sw*_*P2N*_ was in the range of 10^−2^ to 10^−1^. Mulchahy et al. reported the initial fraction of persister cells of *Pseudomonas aeruginosa* isolated from cystic fibrosis patients who succumb to a chronic untreatable *Pseudomonas aeruginosa* infection to be around 10^−4^ to 10^−3^%[59]. On the other hand, longitudinal isolates showed approximately 100-fold higher fractions, which then often contained the *hip* mutation, which increases persister formation, i.e. the switch rate from normal to persister cells *sw*_*N2P*_. In these cystic fibrosis patients, this increased switch rate caused by the *hip* mutation only arose after the persistent infection had been established. This may imply that the switch rate is not a key determinant to establish persistence, similarly to the modeling results pertaining to MRSA persistent bacteremia discussed in this study. Instead, the host-pathogen interaction of persister cells may have an essential role in establishing the persistent infection in cystic fibrosis.

Previous studies modeled the interplay between bacteria, antibiotics, and the immune system to explore optimal antibiotic treatment regimens and the likelihood of emergence of resistance [60,61]. While the models also included subpopulations which acquired antibiotic resistance, the clinical phenomenon of persistence and the factors that may contribute to it, which is the focus of our work, was not explored by these studies.

In this study, while the ensemble approach allowed us to survey a large parameter space, key assumptions were made, and some parameter values or conditions were fixed in an arbitrary manner. To investigate sensitivities of such values to our main conclusion about the key determinant of PB, we repeated the analysis using different values of detection limits of bacteremia (S1 Fig), growth inhibition and killing rate by vancomycin (S2 Fig), initial number of persister cells (S3 Fig), *SAmax* (S4 Fig) and growth rate of normal SA (S5 Fig). Further, in this analysis, we did not explicitly specify a cut-off value to define the extinction of SA, which may affect relapse bacteremia, so we also explored the impact of the values (S6 Fig). Even using these different values, the model reached the same conclusion: the key determinant of PB is the clearance rate of persister cell by immune factors.

In summary, based on these modeling outcomes, we surmise that persistent MRSA bacteremia results from relatively ineffective killing or slow clearance of persister SA by host immunity, even in the context of potent antibiotics. Despite *in vitro* results to the contrary, the clinical significance of switch rates between normal and persister cells is likely to be negligible. For a better understanding of the disease, our modeling results indicate that we need to better understand the pathogen-host interactions of persister MRSAs *in vivo*.

## Methods

### Modeling and simulation

Mathematical model used in the analyses are described in Results section. All the analyses were done in MATLAB version 2017b. The equations were solved numerically by *ode15s*.

### Feature selection and classification for RB and PB

All the models selected were classified into RB or PB and binarized into 0 or 1, respectively, for the subsequent supervised classification modeling. To identify key parameters which discern the two-type of infection, we applied a feature selection method, QPFS. Note that, for classification, we refer to the seven random valued parameters as features. The Pearson correlation statistic was calculated between each of the features and the target class (0 or 1). The same Pearson correlation statistic was also calculated between every pair of features. The absolute values of these correlation statistics yielded a feature relevance vector *F* and redundancy, or cross-correlation matrix *Q*, respectively. From these, a weight vector *x* was calculated that minimizes the quadratic program

$$\underset{x}{\text{min}}\{\frac{1}{2}(1-\alpha )x^{T}Qx-\alpha F^{T}x\}$$ (12)

where a balancing factor *α* was calculated by dividing the mean of *F* by the mean of *Q* to balance the linear (relevance) and quadratic (redundancy) terms. Once the optimal vector of weights, *x*, was identified, we selected top-ranked features to incorporate into a logistic regression model. For the comparison, all parameters and all parameters except for top-ranked features were also applied to the model. Specifically, we modeled the log-odds ratio of the probability of persistent versus resolving as a linear combination of the features with cross-validation. Maximum likelihood estimates of the regression coefficients were achieved using the *fitglm* function in MATLAB, with the link function "*logit*". Receiver Operating Characteristic (ROC) curves were obtained by iterating the log-odds ratio threshold over its full range of possible values, and at each value calculating the true and false positive rates, the ratio of true to total positives, and the ratio of false to total negatives, respectively. As an index of the importance of features incorporated into the model, areas under the ROC curve were calculated for each curve.

### Feature selection and classification for resolving, persistent and relapse bacteremia

The models classified into RB or PB were further divided into with or without relapse bacteremia. Since the number of models for RB with relapse is few, other three classes, RB without relapse, PB without relapse and PB with relapse, were used for the subsequent classification modeling. To select key features of the three classes, QPFS was applied.

The top-ranked features were incorporated into a multinomial logistic regression model with cross-validation. For the comparison, all parameters and all parameters except for top-ranked features were also applied to the model. Since imbalance of class size affects the classification, especially for the classification using irrelevant features, an equal number of data were used by reducing the number of data in major classes to the same number of minor class. As an index of the importance of features incorporated into the model, areas under the ROC curve were calculated for each class.

## Supporting information

S1 Fig Sensitivities of the detection limit of bacteremia. (A) In the main text, we explored the detection limit of bacteremia (Fig 3B) and used 0.1 cfu/a.u. as the limit. The stacked bar plot shows the number of models for resolving (RB) and persistent bacteremia (PB) in each detection limit. “RB -> PB” and”PB -> RB” indicate the models changed to PB and RB, respectively, by altering the value from 0.1 cfu/a.u. (B) The analysis to identify key determinants as shown in Fig 5 was conducted using different values of the detection limit. We conclude that different value of detection limit of bacteremia is less impact on identifying the determinant of persistent bacteremia.
(TIF)

S2 Fig Sensitivities of the pharmacodynamic effects of vancomycin, *d*_*Van*_ and growth inhibition. (A) In the main text, we used 0.3 h^-1^ of *d*_*Van*_ and 95% of growth inhibition as pharmacodynamic effects of vancomycin. The stacked bar plot shows the number of models for resolving (RB) and persistent bacteremia (PB) in each combination of the values. “RB -> PB” and”PB -> RB” indicate the models changed to PB and RB, respectively, by altering the values from those used in the main text. (B) In the absence of the immune system (*in vitro* SA growth model), the number of total SA were simulated with in each combination of the values. Even in the different combination of *d*_*Van*_ and growth inhibition, vancomycin can kill the SA with different strength, except for the combination of *d*_*Van*_ of 0.1 h^-1^ and growth inhibition of 90% (top-left panel). In the condition, the number of SA was static over the time, indicating the condition was an equivalent pharmacological strength to a minimum inhibitory concentration, MIC. Because plasma concentrations of vancomycin in patients is much higher than the MIC, the condition, *d*_*Van*_ of 0.1 and growth inhibition of 90% was considered to be an unlikely condition in clinical. (C,D) The analysis to identify key determinants as shown in Fig 5 was conducted using the different combination of the values. These data indicate that different values of *d*_*Van*_ and growth inhibition within an appropriate range, not ‘MIC’-like condition, is less impact on identifying the determinant of persistent bacteremia. Further, even in the ‘MIC’-like condition, *c*_*P*_ was identified as a major key determinant.
(TIF)

S3 Fig Sensitivities of the initial % of persister cell to total MRSA. (A) In the main text, we assumed that the initial number of persister cells were 0.1% of normal cells. The stacked bar plot shows the number of models for resolving (RB) and persistent bacteremia (PB) in each initial population of persister cells. “RB -> PB” and”PB -> RB” indicate the models changed to PB and RB, respectively, by altering the value from 0.1%. (B) The analysis to identify key determinants as shown in Fig 5 was conducted using the different initial number of persister cells. We conclude that different initial population of persister cells is less impact on identifying the determinant of resolving and persistent bacteremia.
(TIF)

S4 Fig Sensitivities of *SAmax*. (A) In the main text, we assumed that *SAmax* was 1 x 10^8^ cfu/a.u. The stacked bar plot shows the number of models for resolving (RB) and persistent bacteremia (PB) in each value of *SAmax*. “RB -> PB” and”PB -> RB” indicate the models changed to PB and RB, respectively, by altering the value from 1 x 10^8^ cfu/a.u. No difference was observed between the values. (B) In our *in vitro* and *in vivo* mathematical model, saturable growth of normal and persister cells have been expressed by a formulation with *SAmax*. However, in our simulations, both normal and persister cells in *in vitro* and normal cells in *in vivo* never reached to *SAmax* due to the presence of vancomycin (Figs 1 and 6A). Hence, *SAmax* did not affect their simulations at all. On the other hand, persister cells in *in vivo* model also did not showed saturation in their growth in most cases, however, in certain models which showed relapse bacteremia, persister cells could reach to *SAmax* after the repeated cycle of on-off treatments (S4 Fig B). Even in these cases, *SAmax* did not affect the judgement of types of bacteremia in our simulations. Thus, SAmax does not affect the conclusions.
(TIF)

S5 Fig Sensitivities of growth rate of normal SA, *g*_*N*_. In the main text, we used 1.0 h^-1^ of *g*_*N*_. Here, the analysis to identify key determinants as shown in Fig 5 was conducted using 0.5 h^-1^ of *g*_*N*_. We conclude that the slower growth rate of normal SA has little impact on what the determinants of persistent bacteremia are. (A) Violin plots and box plots of the parameter distributions of RB and PB. Dotted lines indicate the range of randomized parameter values. (B) Weights calculated by QPFS methods are shown. (C) The sole ranked feature by QPFS, *c*_*P*_, was used to build a logistic regression model. For the comparison, all parameter and all parameter except for *c*_*P*_ were applied to the classification model. Best accuracy, ROC curve, and area under ROC curve are graphed for each model. (D) Two-dimensional scatter plot for *c*_*P*_ and *sw*_*N2P*_ with probability density for RB and PB.
(TIF)

S6 Fig Sensitivities of cutoff values to define extinction. In the main text, we investigated the relapsing bacteremia which is the absence of an extinction event. Since the mathematical model is deterministic and continuous, it may be desirable to set a cutoff value to define what constitutes an extinction. Here, we analyzed the impact of the cutoff value that defines extinctions. In our simulations, minimum inoculum doses were set between ~100 and ~1000 cfu/au and each inoculum include 0.1% of persister SA. This meant that the cutoff value of extinction should be less than 0.1 cfu/au (= 100 cfu/au x 0.1%). Under an assumption where inoculated persister SA become extinct when they decreased to between 1% and 0.01% of the initial value, we explored the cutoff values from 10^−3^ to 10^−5^ cfu/au. We performed the simulation with 4614 of the selected parameter sets and classified them as shown in Fig 3. During the simulation, when the total SA decreased to less than the cutoff value, then SA were judged as distinct. Bar plots show the number of models classified into resolving bacteremia (RB) with or without relapse and persistent bacteremia (PB) with or without relapse. Only subtle differences were observed in the numbers. Thus, the cutoff value to define extinction does not affect the conclusions.
(TIF)
