# Supplementary figures and images for: Identifying determinants of persistent MRSA bacteremia using mathematical modeling
Source: PLoS Comput Biol. 2019 Jul 11;15(7):e1007087. doi: 10.1371/journal.pcbi.1007087 (PMC6622483; doi:10.1371/journal.pcbi.1007087)

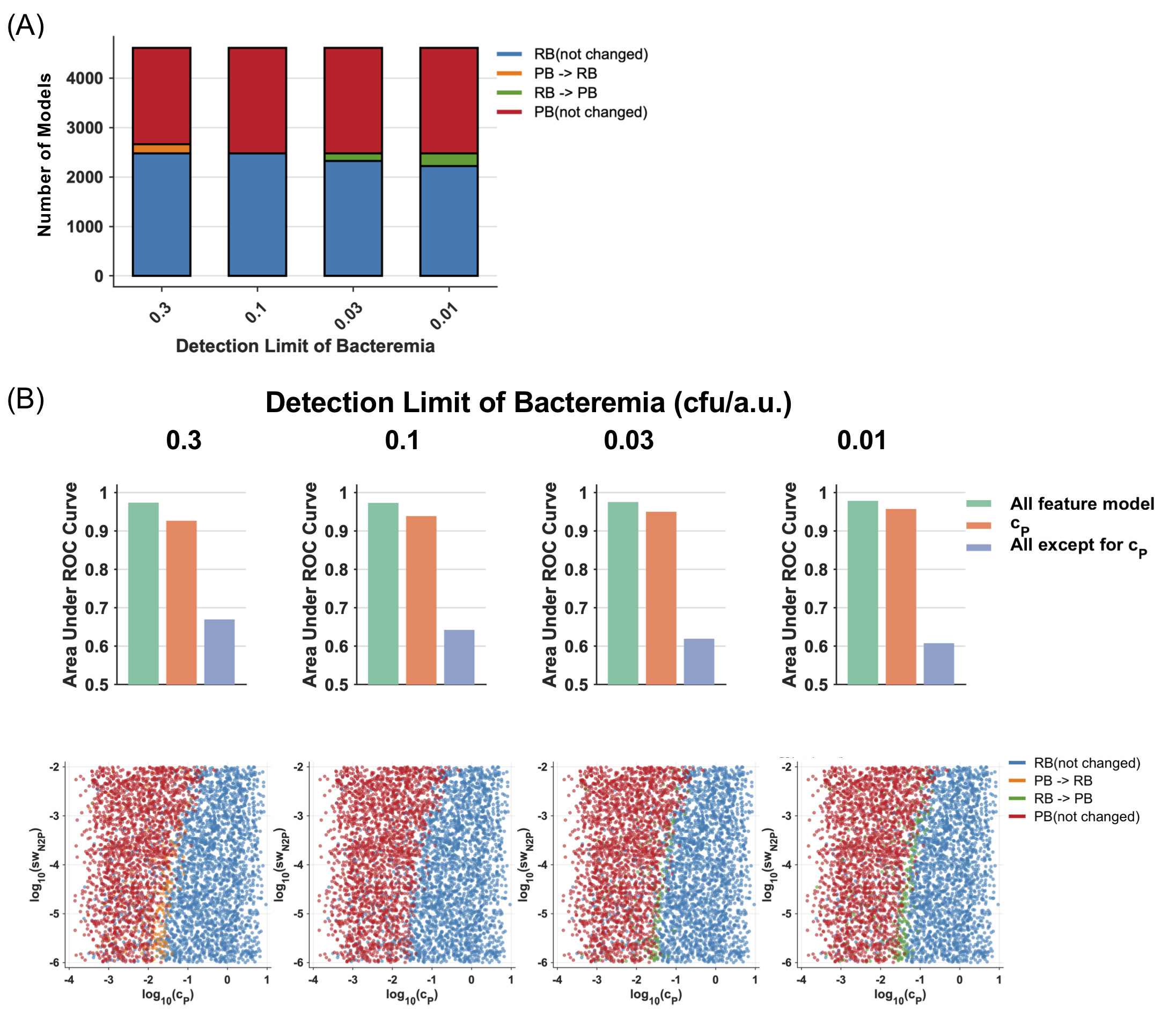

Supplement: S1 Fig — (A) In the main text, we explored the detection limit of bacteremia (Fig 3B) and used 0.1 cfu/a.u. as the limit. The stacked bar plot shows the number of models for resolving (RB) and persistent bacteremia (PB) in each detection limit. “RB -> PB” and”PB -> RB” indicate the models changed to PB and RB, respectively, by altering the value from 0.1 cfu/a.u. (B) The analysis to identify key determinants as shown in Fig 5 was conducted using different values of the detection limit. We conclude that different value of detection limit of bacteremia is less impact on identifying the determinant of persistent bacteremia. (TIF) [file pcbi.1007087.s001.tif]

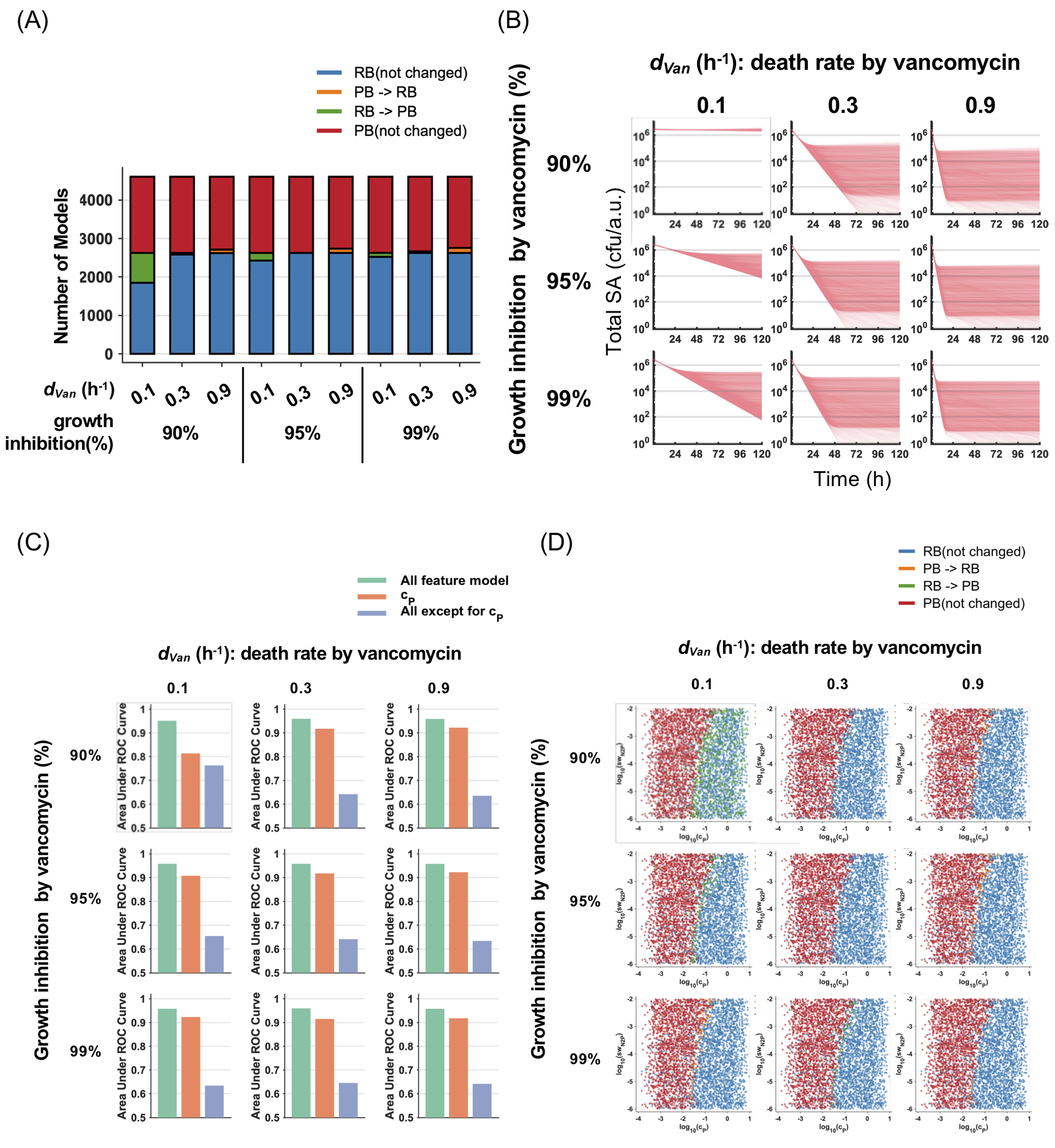

Supplement: S2 Fig — (A) In the main text, we used 0.3 h-1 of dVan and 95% of growth inhibition as pharmacodynamic effects of vancomycin. The stacked bar plot shows the number of models for resolving (RB) and persistent bacteremia (PB) in each combination of the values. “RB -> PB” and”PB -> RB” indicate the models changed to PB and RB, respectively, by altering the values from those used in the main text. (B) In the absence of the immune system (in vitro SA growth model), the number of total SA were simulated with in each combination of the values. Even in the different combination of dVan and growth inhibition, vancomycin can kill the SA with different strength, except for the combination of dVan of 0.1 h-1 and growth inhibition of 90% (top-left panel). In the condition, the number of SA was static over the time, indicating the condition was an equivalent pharmacological strength to a minimum inhibitory concentration, MIC. Because plasma concentrations of vancomycin in patients is much higher than the MIC, the condition, dVan of 0.1 and growth inhibition of 90% was considered to be an unlikely condition in clinical. (C,D) The analysis to identify key determinants as shown in Fig 5 was conducted using the different combination of the values. These data indicate that different values of dVan and growth inhibition within an appropriate range, not ‘MIC’-like condition, is less impact on identifying the determinant of persistent bacteremia. Further, even in the ‘MIC’-like condition, cP was identified as a major key determinant. (TIF) [file pcbi.1007087.s002.tif]

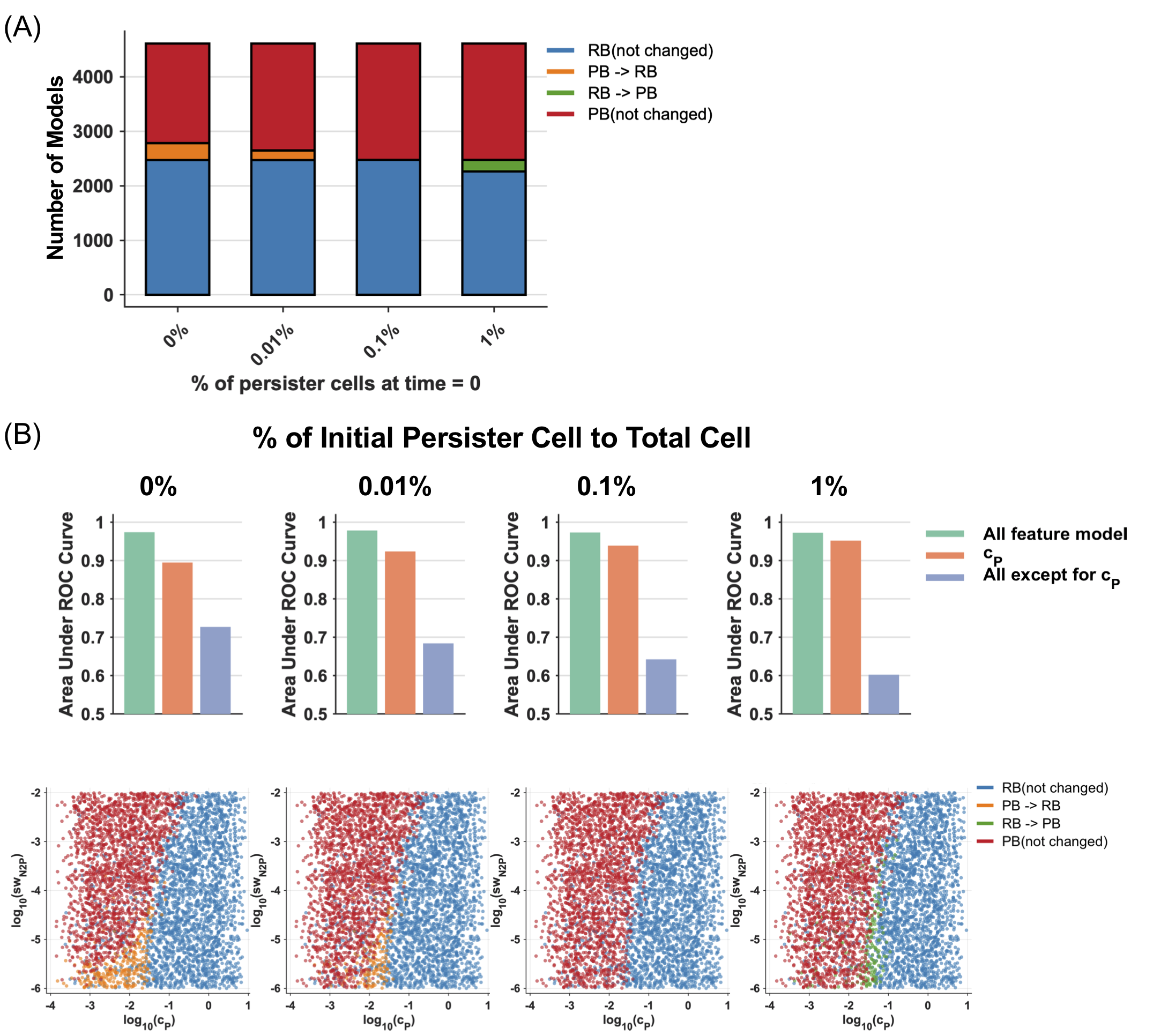

Supplement: S3 Fig — (A) In the main text, we assumed that the initial number of persister cells were 0.1% of normal cells. The stacked bar plot shows the number of models for resolving (RB) and persistent bacteremia (PB) in each initial population of persister cells. “RB -> PB” and”PB -> RB” indicate the models changed to PB and RB, respectively, by altering the value from 0.1%. (B) The analysis to identify key determinants as shown in Fig 5 was conducted using the different initial number of persister cells. We conclude that different initial population of persister cells is less impact on identifying the determinant of resolving and persistent bacteremia. (TIF) [file pcbi.1007087.s003.tif]

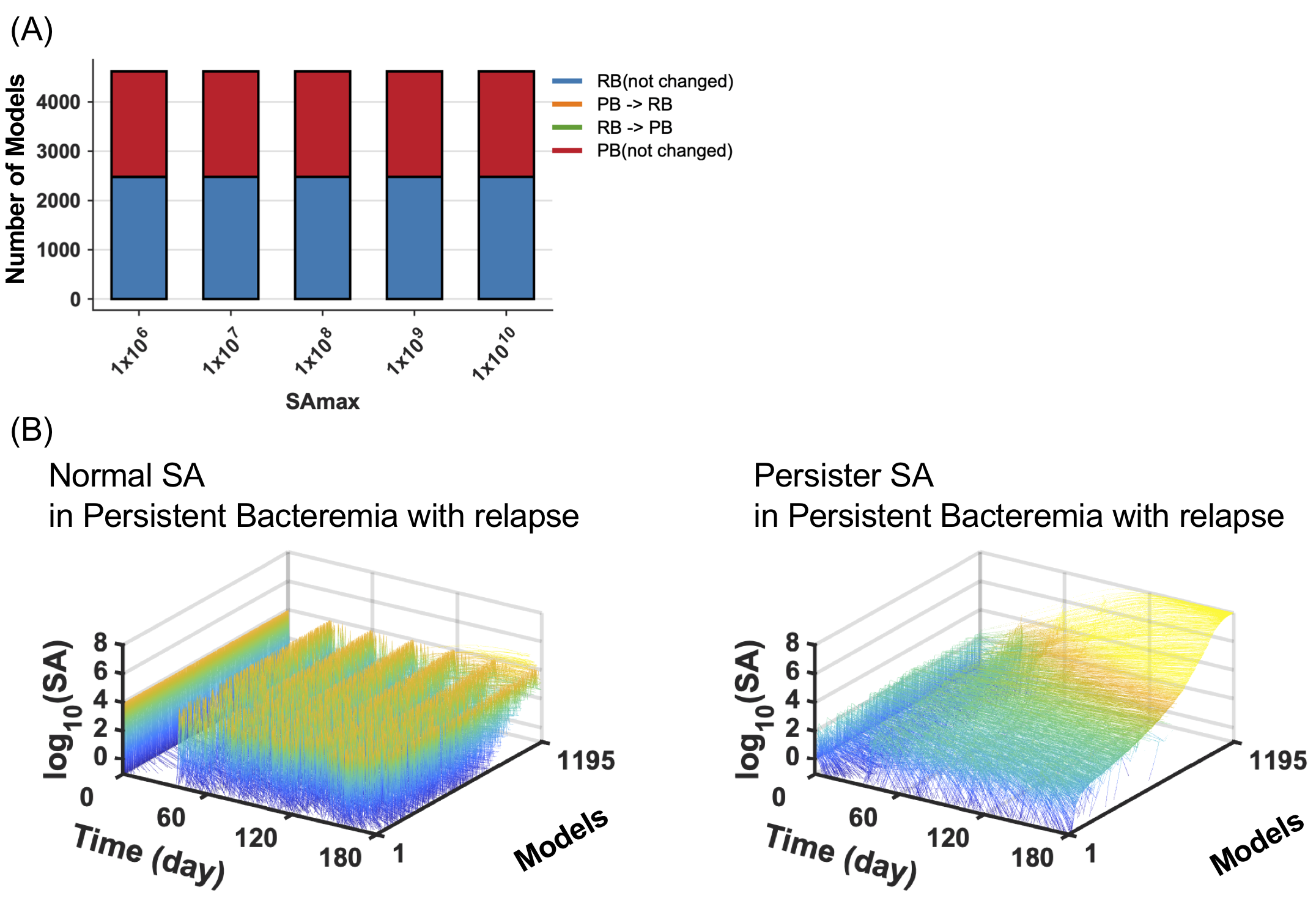

Supplement: S4 Fig — (A) In the main text, we assumed that SAmax was 1 x 10^8^ cfu/a.u. The stacked bar plot shows the number of models for resolving (RB) and persistent bacteremia (PB) in each value of SAmax. “RB -> PB” and”PB -> RB” indicate the models changed to PB and RB, respectively, by altering the value from 1 x 10^8^ cfu/a.u. No difference was observed between the values. (B) In our in vitro and in vivo mathematical model, saturable growth of normal and persister cells have been expressed by a formulation with SAmax. However, in our simulations, both normal and persister cells in in vitro and normal cells in in vivo never reached to SAmax due to the presence of vancomycin (Figs 1 and 6A). Hence, SAmax did not affect their simulations at all. On the other hand, persister cells in in vivo model also did not showed saturation in their growth in most cases, however, in certain models which showed relapse bacteremia, persister cells could reach to SAmax after the repeated cycle of on-off treatments (S4 Fig B). Even in these cases, SAmax did not affect the judgement of types of bacteremia in our simulations. Thus, SAmax does not affect the conclusions. (TIF) [file pcbi.1007087.s004.tif]

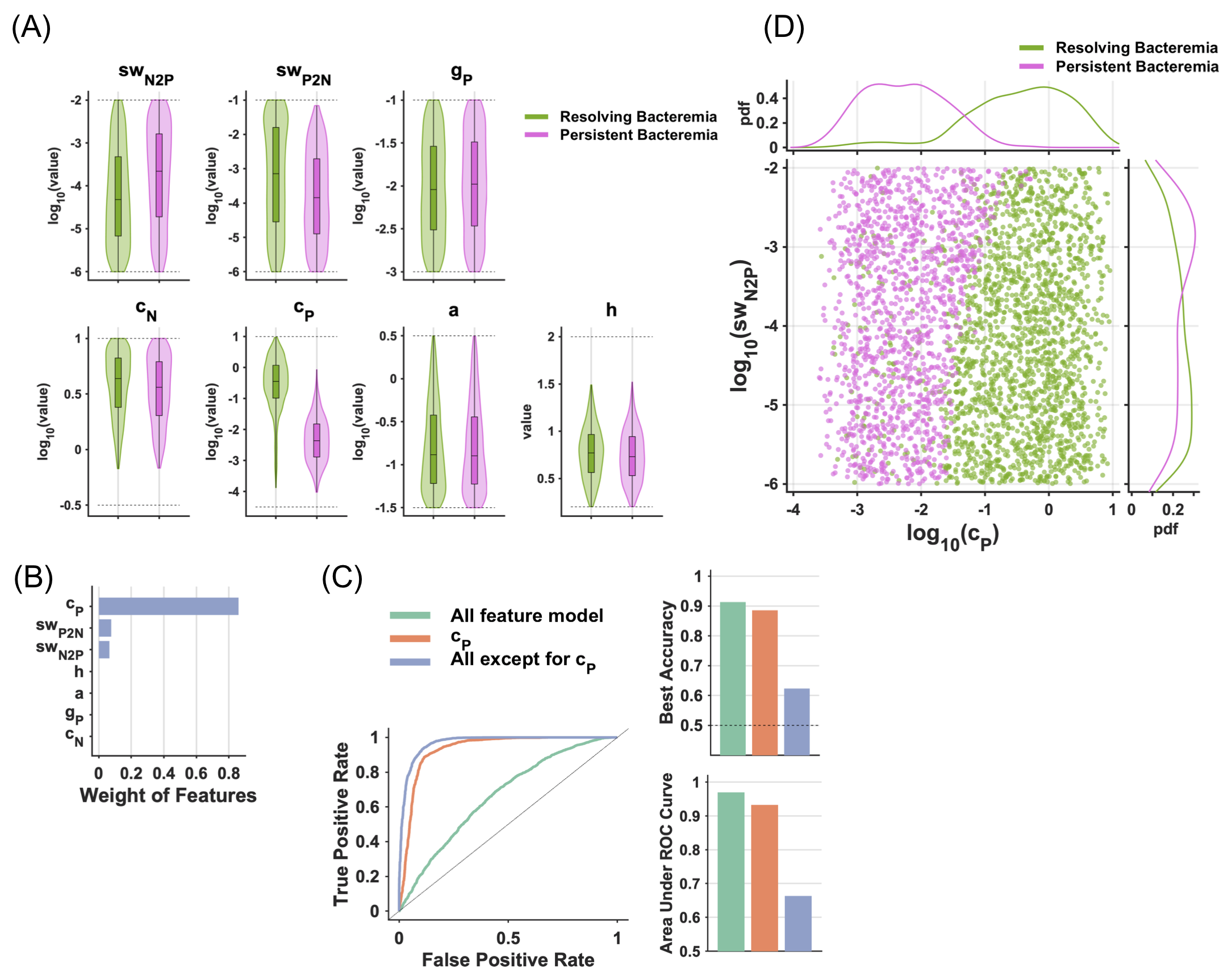

Supplement: S5 Fig — Here, the analysis to identify key determinants as shown in Fig 5 was conducted using 0.5 h-1 of gN. We conclude that the slower growth rate of normal SA has little impact on what the determinants of persistent bacteremia are. (A) Violin plots and box plots of the parameter distributions of RB and PB. Dotted lines indicate the range of randomized parameter values. (B) Weights calculated by QPFS methods are shown. (C) The sole ranked feature by QPFS, cP, was used to build a logistic regression model. For the comparison, all parameter and all parameter except for cP were applied to the classification model. Best accuracy, ROC curve, and area under ROC curve are graphed for each model. (D) Two-dimensional scatter plot for cP and swN2P with probability density for RB and PB. (TIF) [file pcbi.1007087.s005.tif]

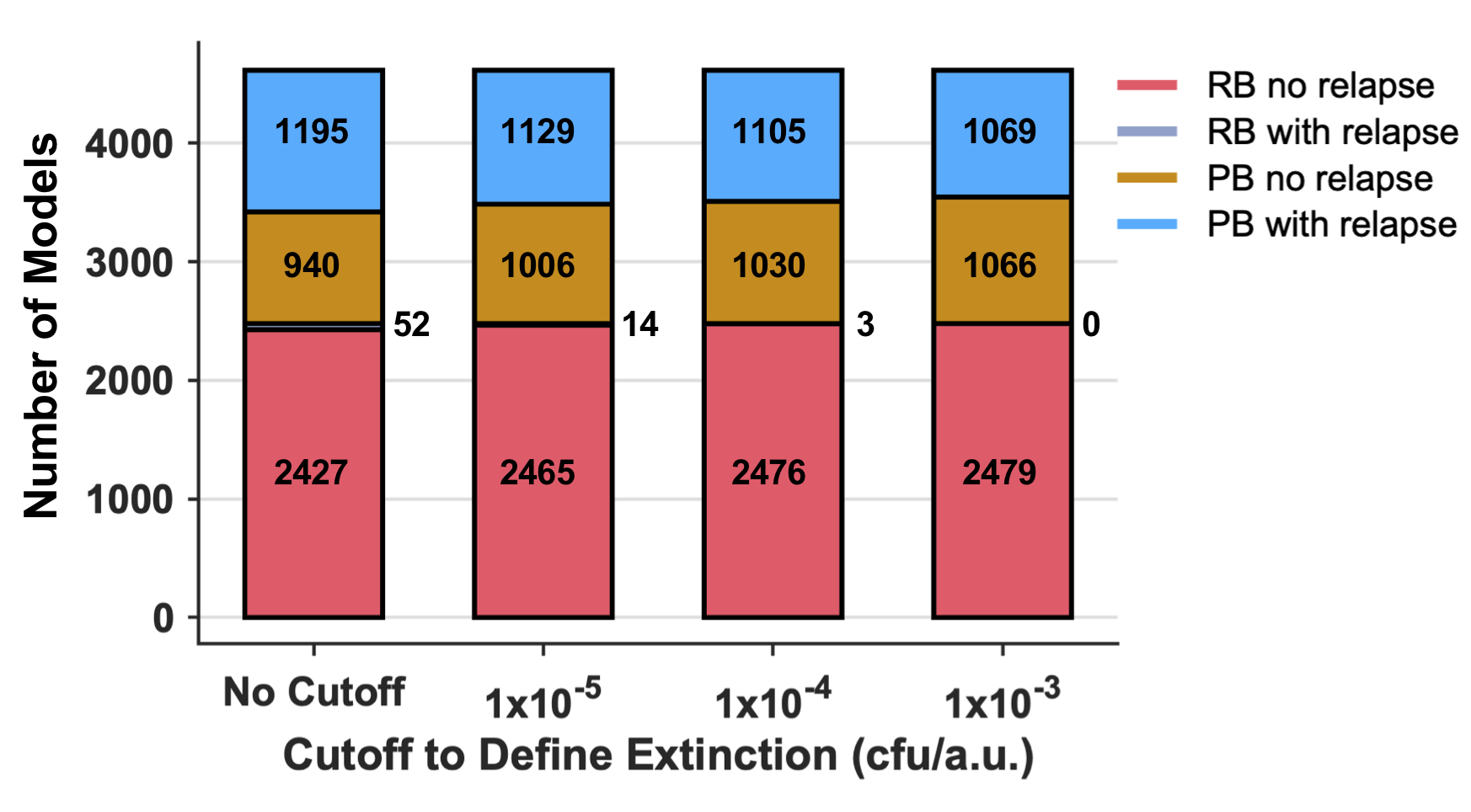

Supplement: S6 Fig — In the main text, we investigated the relapsing bacteremia which is the absence of an extinction event. Since the mathematical model is deterministic and continuous, it may be desirable to set a cutoff value to define what constitutes an extinction. Here, we analyzed the impact of the cutoff value that defines extinctions. In our simulations, minimum inoculum doses were set between ~100 and ~1000 cfu/au and each inoculum include 0.1% of persister SA. This meant that the cutoff value of extinction should be less than 0.1 cfu/au (= 100 cfu/au x 0.1%). Under an assumption where inoculated persister SA become extinct when they decreased to between 1% and 0.01% of the initial value, we explored the cutoff values from 10−3 to 10−5 cfu/au. We performed the simulation with 4614 of the selected parameter sets and classified them as shown in Fig 3. During the simulation, when the total SA decreased to less than the cutoff value, then SA were judged as distinct. Bar plots show the number of models classified into resolving bacteremia (RB) with or without relapse and persistent bacteremia (PB) with or without relapse. Only subtle differences were observed in the numbers. Thus, the cutoff value to define extinction does not affect the conclusions. (TIF) [file pcbi.1007087.s006.tif]
